# HDAC3 inhibition ameliorates spinal cord injury by immunomodulation

**DOI:** 10.1038/s41598-017-08535-4

**Published:** 2017-08-17

**Authors:** Tomoharu Kuboyama, Shalaka Wahane, Yong Huang, Xiang Zhou, Jamie K. Wong, Andrew Koemeter-Cox, Michael Martini, Roland H. Friedel, Hongyan Zou

**Affiliations:** 1 Fishberg Department of Neuroscience, Friedman Brain Institute, Icahn School of Medicine at Mount Sinai, New York, New York 10029 USA; 2 Department of Neurosurgery, Friedman Brain Institute, Icahn School of Medicine at Mount Sinai, New York, New York 10029 USA; 3 Division of Neuromedical Science, Institute of Natural Medicine, University of Toyama, Toyama, 930-0194 Japan; 4 Present Address: Tisch MS Research Center of New York, New York, New York 10019 USA; 5 Present Address: Alzheimer’s Drug Discovery Foundation New York, New York, 10019 USA

## Abstract

Following spinal cord injury (SCI), the innate immune response of microglia and infiltrating macrophages clears up cellular debris and promotes tissue repair, but it also inflicts secondary injury from inflammatory responses. Immunomodulation aimed at maximizing the beneficial effects while minimizing the detrimental roles of the innate immunity may aid functional recovery after SCI. However, intracellular drivers of global reprogramming of the inflammatory gene networks in the innate immune cells are poorly understood. Here we show that SCI resulted in an upregulation of histone deacetylase 3 (HDAC3) in the innate immune cells at the injury site. Remarkably, blocking HDAC3 with a selective small molecule inhibitor shifted microglia/macrophage responses towards inflammatory suppression, resulting in neuroprotective phenotypes and improved functional recovery in SCI model. Mechanistically, HDAC3 activity is largely responsible for histone deacetylation and inflammatory responses of primary microglia to classic inflammatory stimuli. Our results reveal a novel function of HDAC3 inhibitor in promoting functional recovery after SCI by dampening inflammatory cytokines, thus pointing towards a new direction of immunomodulation for SCI repair.

## Introduction

Adult mammalian neurons face multiple barriers for functional recovery after CNS injury. Earlier research has focused on inhibitory molecules of myelin debris and astroglial scar^1–4^; far less is understood of the inflammatory immune response, which constitutes another hurdle for neural repair. Spinal cord injury (SCI) triggers a multiphasic immune response. Shortly after injury, resident microglia are activated, while blood-borne monocytes begin to infiltrate the injury site to differentiate into phagocytic macrophages, which become largely indistinguishable from activated microglia by morphology and cell-surface markers^5^. Together, they constitute the innate immunity that clears up cellular debris^6,7^, but they also release inflammatory cytokines, proteases, and free radicals to fuel secondary injury^8^. Hence, microglia/macrophages play a dual role in SCI repair^9^.

A prevailing concept of macrophage activation is the so-called macrophage polarization along the M1 (pro-inflammatory) and M2 (anti-inflammatory) spectrum, which helps to simplify the conceptualization of the divergent functions of the innate immune response after injury. The M1/M2 model was originally developed based on *in vitro* studies of exposing isolated macrophages to purified stimuli: M1 phenotype is induced by classic inflammatory cytokine interferon-γ (IFNγ) in conjunction with lipopolysaccharide (LPS), an activator of the toll-like receptor; while M2 phenotype is induced by Th2 cytokines such as interleukin 4 (IL-4) and IL-13^10^. Accumulating evidence, however, shows a disconnect of the M1/M2 polarization model with *in vivo* functionalities of macrophages^11^. For instance, recent studies found induction of a combination of M1- and M2-associated genes in SCI and traumatic brain injury (TBI)^12–14^. Furthermore, single cell RNA-Seq analysis revealed that microglia frequently co-express both canonical markers of M1 and M2, or neither, in single cells, and that expression of polarization markers does not predict the expression of other polarization genes^15^. Thus, *in vivo* gene signatures of microglia/macrophages in specific CNS injury settings remain to be clarified. In contrast, contextual factors that govern the various reactive states of microglia/macrophages after CNS injury have increasingly come to light. For instance, after SCI, the classic inflammatory cytokine IFNγ is upregulated, whereas the levels of Th2 cytokines, IL-4 and IL-13, remain low^14^. Nevertheless, far less is understood of the transcriptional machinery that is responsible for regulating the distinct gene networks of different activation states of microglia/macrophages in response to specific contextual stimuli after SCI.

Transcriptional signatures of different cell types are shaped by unique chromatin landscapes. As macrophage activation is associated with global reprogramming of gene expression, chromatin structure alterations as modulated by epigenetic factors would set the stage for access of transcription factors to a large repertoire of genomic loci in a coordinated fashion. In agreement with this notion, histone acetylation has been implicated in the regulation of macrophage responses^16,17^. Histone acetylation is regulated by the opposing activities of histone acetyltransferases (HATs) and histone deacetylases (HDACs). Among the four classes of HDACs, class I HDACs (1, 2, 3, and 8) are predominantly nuclear enzymes, acting as transcriptional co-repressors at specific genomic loci^18^. The specific HDAC isoforms that are mainly responsible for regulating the immune reaction after SCI are unknown. Here, we identified a robust upregulation of HDAC3 in innate immune cells after SCI. Importantly, administering a small-molecule inhibitor RGFP966 that specifically targets HDAC3 resulted in global suppression of inflammatory cytokines at the injury milieu, leading to neuroprotective phenotypes and improved functional recovery after SCI.

## Results

### Microglia/macrophages upregulate HDAC3 after spinal cord injury

**Figure 1:**
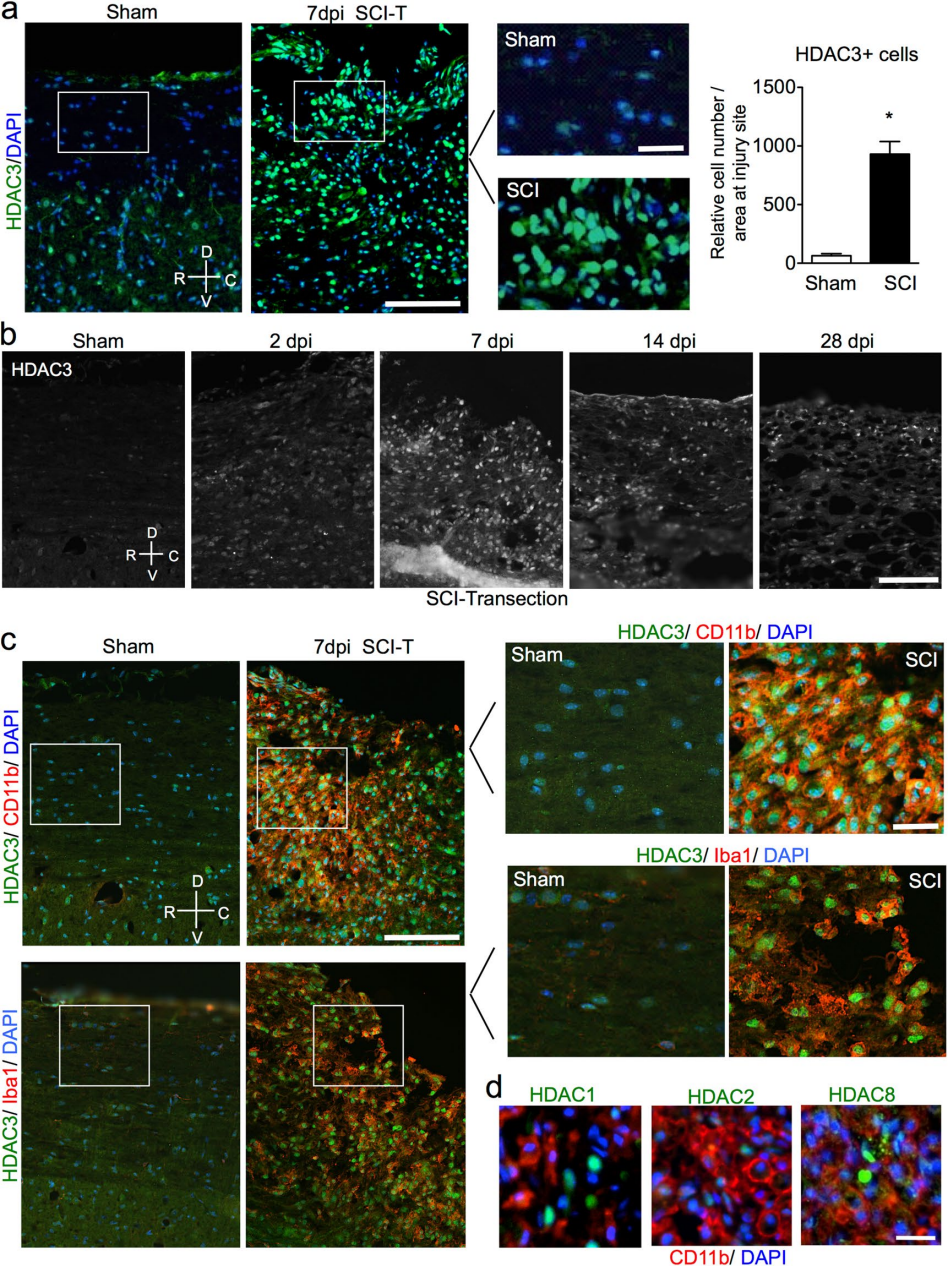
Upregulation of HDAC3 in microglia/macrophages after SCI. (**a**) Representative sagittal immunohistochemistry images and quantification demonstrate upregulation of HDAC3 (green) at lesion center 7 days post injury (7 dpi) by T8 dorsal column transection (SCI-T). DAPI nuclear counterstaining highlights the increased cellularity at lesion core. Enlarged images of boxed areas are shown on the right. Quantification of the number of HDAC3+ cells at the injury site is shown on the right: *p = 0.015, parametric slit-plot ANOVA. 3 pairs of mice were examined, with 3 representative images quantified for each animal. (**b**) Representative immunohistochemistry images of HDAC3 at different time points after SCI-T as compared to sham. (**c**) Representative immunofluorescent images of co-immunolabeling show a large overlap of HDAC3+ cells (green) and CD11b+ or Iba1+ immune cells (red) at the injury site at 7 days after SCI-T. Enlarged images of boxed areas are shown on the right. (**d**) Immunohistochemistry images for CD11b (red) and different class I HDACs (green) at 7 days after SCI-T. No significant overlaps were detected between these HDACs and CD11b. Orientation: R: Rostral, C: Caudal, D: Dorsal, V: Ventral. Scale bars: 100 µm (**a**,**b**,**c**), 25 µm (**d**, enlarged images in **a**,**c**).

We first examined expression dynamics of class I HDACs after SCI by immunohistochemistry (IHC). In two defined *in vivo* models of SCI –dorsal column transection and contusion injury–, we found a robust induction of HDAC3 at the lesion center at 7 days post-injury (dpi) (Fig. 1a and Supplementary Fig. 1). In comparison, HDAC2 and 8 were upregulated more prominently at lesion borders, whereas HDAC1 was induced in both lesion core and lesion periphery, including axon tracts (Supplementary Fig. 1). Temporal analysis demonstrated that HDAC3 upregulation was detectable at 2 dpi, peaked at 7 dpi, and was sustained but slighted waned at 14 and 28 dpi (Fig. 1b). When overlaid with CD11b, a cell-surface marker for microglia and monocyte-derived inflammatory macrophages in the CNS, only HDAC3, but no other class I HDAC, was markedly upregulated in the nuclei of CD11b^+^ cells (Fig. 1c,d). Thus, different class I HDACs appear to be differentially regulated in different cell types after SCI. Co-immunostaining for HDAC3 and Iba1, another well-established marker for activated microglia and macrophages, confirmed HDAC3 induction in the innate immune cells, with majority of Iba1^+^ cells at the injury site displaying prominent nuclear HDAC3 immunosignals, similar to that in CD11b^+^ cells (Fig. 1c).

**Figure 2:**
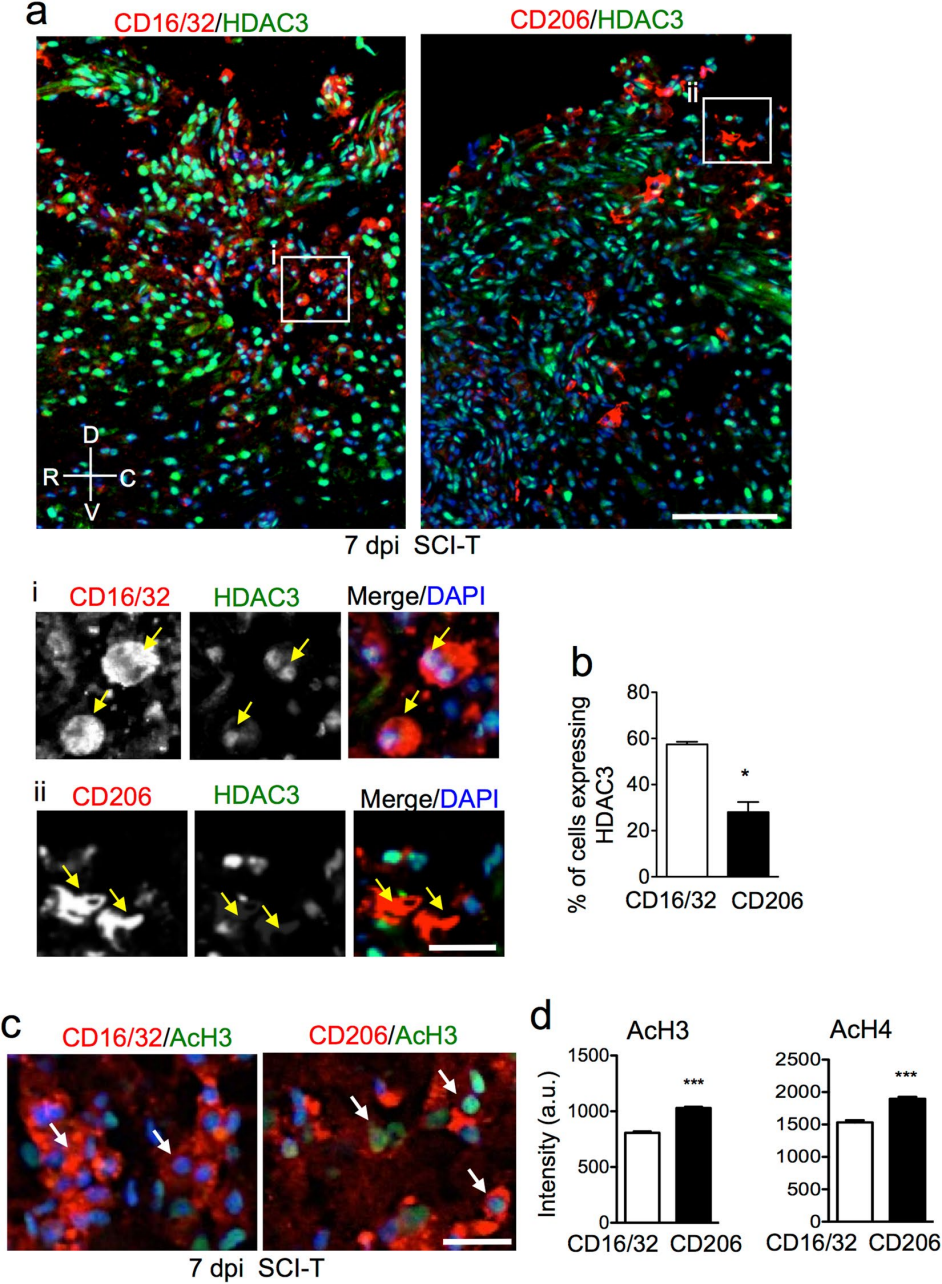
Upregulation of HDAC3 correlates with decreased histone acetylation level. (**a**,**b**) Representative images and quantification of co-immunolabeling for cell surface markers (red) and HDAC3 (green) at the lesion center 7 days post T8 dorsal column transection (SCI-T). Enlarged images of boxed areas (i and ii) are shown below. Arrows point to nuclear HDAC3 immunoreactivity. *p = 0.024, parametric split-plot ANOVA, n = 3 pairs of mice, with three representative image for each animal. (**c**,**d**) Representative images and quantification of co-immunolabeling demonstrate that CD206+ macrophages (red) display higher levels of AcH3 (green) than CD16/32+ macrophages (red), as well as higher levels of AcH4 (**d**). For AcH3 levels, 3029 CD16/32+ cells and 6245 CD206+ cells from 3 animals, with 3 images from each animal were quantified. For AcH4, 1687 CD16/32+ cells and 2305 CD206+ cells from 3 animals, with 3 images from each animal were quantified. ***p < 0.001, Mann Whitney test. Orientation: R: Rostral, C: Caudal, D: Dorsal, V: Ventral. Scale bars: 100 µm (**a**), 25 µm (insets i and ii), 20 µm (**c**).

The increased nuclear accumulation of HDAC3 in CD11b^+^ and Iba1^+^ immune cells after SCI raises the tantalizing possibility that it may function as an epigenetic regulator of the inflammatory responses of microglial/macrophages after SCI. We performed co-immunostaining with well-known cell surface markers of macrophages of different activation states: CD16/32 to identify macrophages in an inflammatory state, and CD206 to identify macrophages in an anti-inflammatory/pro-repair state^19^. IHC showed that HDAC3 induction occurred more prominently in CD16/32^+^ than CD206^+^ macrophages: at 7 dpi after dorsal column transection, nuclear HDAC3 expression was found in close to 60% of CD16/32^+^ cells as compared to 30% of CD206^+^ cells (p = 0.024, parametric Split-plot ANOVA, Fig. 2a,b). The different extent of HDAC3 induction in different subpopulations not only demonstrates heterogeneity among microglia/macrophages in response to activating stimuli in SCI, but may also reflect a dynamic process of the innate immune response with constant influx of macrophages arriving at different time points and settling in different niches in a changing injury microenvironment.

Previous genome-wide analyses revealed that HDAC3 functions as a part of transcriptional repressor complex at target genes to remove acetyl moieties from histone 3 lysine 9 (H3K9Ac)^20^. We therefore compared global levels of histone acetylation in different microglia/macrophage subpopulations at the injury site by IHC. We found that CD16/32^+^ immune cells displayed lower levels of acetylated histones H3 and H4 (AcH3 and AcH4) than CD206^+^ cells (Fig. 2c,d). Hence, upregulation of HDAC3 in the inflammatory CD16/32^+^ cells during the innate immune response to SCI correlated with a global reduction of histone acetylation in this subpopulation.

### HDAC3 inhibition results in global inflammatory suppression

**Figure 3:**
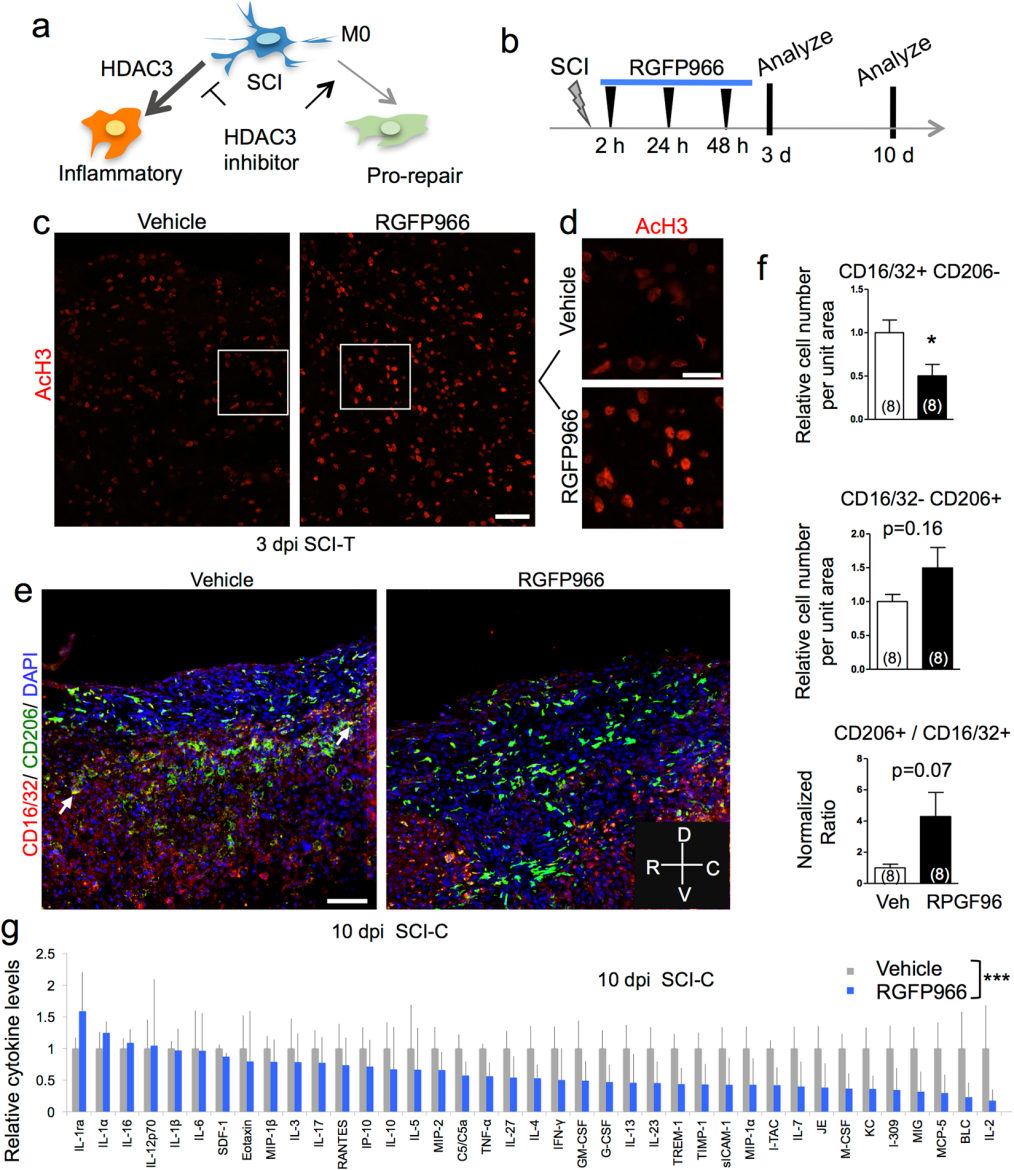
HDAC3 inhibition changes functional state of innate immune cells and leads to global inflammatory suppression in SCI. (**a**) Diagram of divergent macrophage responses and hypothesized role of HDAC3. M0: homeostatic macrophages. (**b**) Experimental scheme of HDAC3 inhibitor treatment after SCI. Three i.p. injections of RGFP966 were delivered. (**c**,**d**) Representative IHC images showing increased AcH3 levels at the injury site with RPGF966 treatment at 3 dpi after T8 dorsal column transection (SCI-T). Enlarged images in the boxed area are shown in (**d**). (**e**,**f**) Representative immunofluorescent images and quantification of the numbers of CD16/32 immune cells (green), CD206 immune cells (red), and the ratio of the two subpopulations at the injury site at 10 dpi after contusion injury (SCI-C). DAPI was used for nuclear counterstaining. Arrow: example of double positive cell (CD16/32+ CD206+). n = 8 mice per group, with 2–4 representative images averaged for each animal. *p < 0.05, Unpaired Student’s t-test. (**g**) Relative cytokine/chemokine levels as determined by cytokine array using lysates of injured spinal cord from vehicle or RFGP966-treated animals at 10 dpi after contusion SCI. ***p < 0.001, two-way ANOVA with Bonferroni post hoc correction (n = 4 pairs of mice). Orientation: R: Rostral, C: Caudal, D: Dorsal, V: Ventral. Scale bar: 50 µm (**c**), 25 µm (**d**), and 100 µm (**e**).

To directly test the hypothesis that HDAC3 may function as a driver for the inflammatory responses of microglia/macrophages after SCI (Fig. 3a), we took advantage of a highly selective HDAC3 inhibitor, RGFP966, which displays >200-fold selectivity over other HDACs^21^. Notably, RGFP966 can penetrate the blood brain barrier (BBB) and distribute efficiently in the CNS^21,22^. RGFP966 or vehicle was administered to animals by three intraperitoneal (i.p.) injections at 2, 24, and 48 hr after a T8 contusion injury (Fig. 3b). An earlier pharmacokinetic study showed that a maximum drug concentration of RGFP966 was observed in the CNS at 15 min after i.p. injection, reaching ~110 fold of the HDAC3 IC_50_ value of 0.08 µM, and at 2 hr after injection, the drug level persisted with over 8 fold of the HDAC3 IC_50_ value^22^.

We first confirmed the *in vivo* effect of RGFP966 on global levels of histone acetylation after SCI. We examined spinal cord at 3 dpi, an early time point, to assess the immediate effect of RGFP966 administered at 2, 24 and 48 h after transection SCI. Indeed, the global AcH3 levels appeared higher at the injury site in RGFP966 treated animals than controls (Fig. 2c,d), consistent with earlier pharmacokinetic data on efficient CNS penetrance of RGFP966 and the known enzymatic function of HDAC3^22^. To focus on the immune cells, we tested the effect of RGFP966 on global histone acetylation in a reporter line, INTACT (isolation of nuclei tagged in specific cell types), which enables Cre-dependent conditional expression of GFP-tagged nuclear membrane protein Sun-1^23^ (Supplementary Fig. 2). The INTACT mice were crossed to a tamoxifen-inducible, myeloid specific Cx3cr1-CreER line^24^. For visual identification of innate immune cells at the injury site, we administered tamoxifen at 3 d and 1 d before the SCI to label microglia and infiltrating macrophages (Supplementary Fig. 2). IHC images and quantification demonstrated that RGFP966 treatment resulted in increased average AcH3 levels in GFP^+^ immune cells at 3 dpi (p < 0.001, Mann Whitney test, Supplementary Fig. 2). At 10 days after SCI, there was a persistent trend of increase in the average AcH3 levels in Iba1^+^ cells with RGFP966 treatment (Supplementary Fig. 2). Notably, AcH3 levels in individual Iba1^+^ cells appeared heterogeneous, reflective of the diversity of the macrophage subpopulations during the multiphasic immune response.

We then further examined the spinal cord at 10 dpi, a time point representing peak phagocytic activity of microglia/macrophages after SCI. We first tested whether inhibition of HDAC3 activity by RGFP966 would lead to altered influx of macrophages and proliferation of the innate immune cells after SCI. We compared the number and spatial distribution of Iba1^+^ and CD11b^+^ immune cells at the injury site, but found no overt differences between the vehicle- and RGFP966-treated cohorts in either transection or contusion SCI models (Supplementary Fig. 3). This suggests that proliferation of resident microglia and the influx of monocyte-derived macrophages were not significantly affected by HDAC3 inhibition. Next, we compared microglia/macrophage subpopulations by co-labeling for CD16/32 and CD206. There was a notable double positive (CD16/32^+^ CD206^+^) population in the injured spinal cord tissues (Fig. 3e), which echoes single cell RNA-Seq analysis showing simultaneous expression of M1- and M2-like profiles in brain macrophages after traumatic CNS injury^15^. We subsequently focused on the immune cells expressing only single markers. We found that the number of CD16/32^+^ CD206^−^ cells at the injury site at 10 dpi after spinal cord contusion was significantly decreased in RGFP966-treated animals vs. controls (p = 0.025, Fig. 3f). In comparison, there was a trend of increase in the number of CD206^+^ CD16/32^−^ cells in the RGFP966 cohort (p = 0.16). Hence, HDAC3 inhibition by RGFP966 resulted in an increased ratio of CD206^+^ over CD16/32^+^ cells at the injury site (p = 0.07). Similar changes in the number and ratio of the two subpopulations of microglia/macrophages were observed with RGFP966 treatment in the dorsal column transection SCI model (Supplementary Fig. 3), indicating a broad efficacy of RGFP966 in influencing microglia/macrophage responses in different SCI models.

To further test the model that HDAC3 inhibition may change the inflammatory milieu at the injury site, we compared cytokine levels in spinal cord tissues using a cytokine/chemokine antibody array. Strikingly, HDAC3 inhibition with RGFP966 after SCI resulted in a broad suppression of cytokines, chemokines, and acute phase proteins when examined at 10 dpi after contusion injury. Specifically, 32 out of the 39 proteins measured showed decreased levels in the RGFP966-treated cohort (p < 0.001, two-way ANOVA) (Fig. 3g), including classic inflammatory cytokines such as TNFα and IFNγ. Four cytokines (IL-16, IL-12p70, IL-1β, and IL-6) showed no detectable changes between the two cohorts, whereas only two cytokines, IL-1ra (Interleukin-1 receptor antagonist) and IL-1α, showed a modest increase in RGFP966-treated animals relative to controls. Notably, IL-1ra is a natural inhibitor of the pro-inflammatory effect of IL-1β^25^. It is also noteworthy that many cytokines/chemokines associated with the M2-like phenotype, such as IL-4, 10, and 13, were also repressed, indicating that HDAC3 inhibition resulted in a broad inflammatory suppression, distinct from a simple shift between the two polarized states. Our results further echo the current view to move away from the tenet of M1/M2 polarization for characterizing the diverse *in vivo* functional states of microglia/macrophages.

### Inhibition of HDAC3 improves functional recovery after SCI

Since microglia/macrophages can exert pro-repair functions^14^, we next assessed the impact of HDAC3 inhibition on functional recovery by behavioral assays in the contusion SCI model. RGFP966 was administered at 2, 24 and 48 hr after SCI, and locomotor recovery was measured daily using the Basso mouse scale (BMS)^26^ and the Toyama mouse score (TMS)^27^. The 0–8 point BMS score provides a measure of hind limb locomotor recovery, with score 3 reflecting the threshold at which an animal can support its own body weight, and scores beyond 3 reflecting mainly improved stepping frequency and consistency. TMS is a modified score system with an emphasis on hindlimb body trunk support. TMS takes into consideration ankle movement, as well as movement of knees, thighs, and toes, which are not observed in the BMS. The TMS provides lower variation and higher sensitivity relative to the BMS^27^.

**Figure 4:**
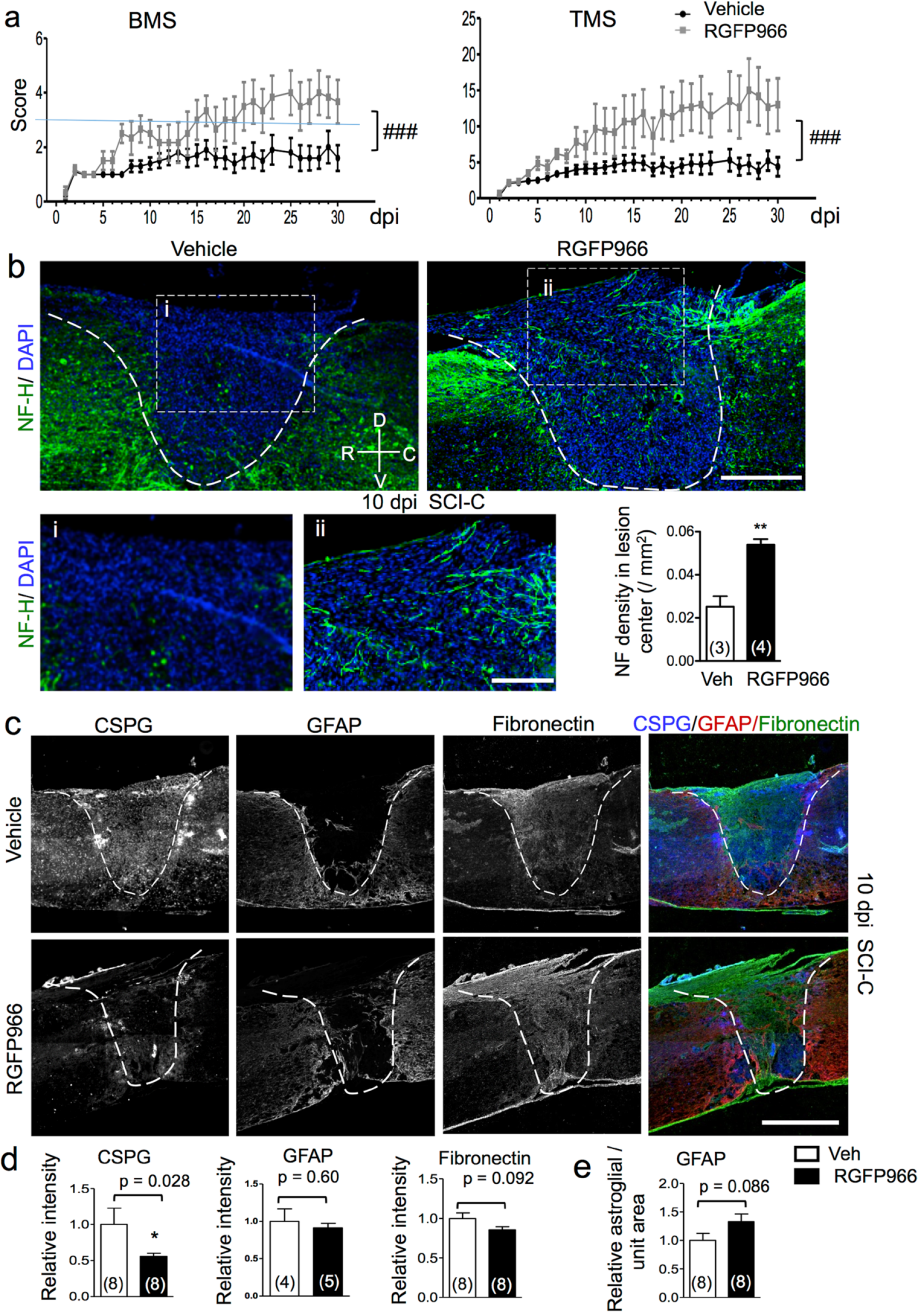
HDAC3 inhibition improves functional recovery and enhances neuroprotective phenotypes after SCI. (**a**) Behavioral assays demonstrate improved and sustained functional recovery by both BMS and TMS scores up to 30 dpi after contusion injury. Blue line in the BMS score denotes weight-bearing threshold. Repeated measures, two-way ANOVA with Bonferroni post hoc correction. ^###^p < 0.001. n = 10 for vehicle and n = 6 for RGFP966 cohort. (**b**) Representative images and quantification show increased density of axon fibers, identified by NF-H immunolabeling (green), in lesion center at 10 dpi after contusion injury (SCI-C). Lesion border is indicated by dashed lines. DAPI was used for nuclear counterstaining. Enlarged images of boxed areas are shown below. n = 3 mice for vehicle and 4 mice for RGFP966 cohort, with 1–4 representative images averaged for each animal. **p < 0.01, unpaired Student’s t-test. (**c**–**e**) Representative images and quantification of SCI scar constituents at 10 dpi after contusion: CSPG (blue), GFAP (red), and Fibronectin (green). Lesion border is indicated by dashed lines. The number of animals quantified is shown inside the bar graph, with 2–5 representative image averaged for each animal. Quantification showed a decrease in CSPG with RGFP966 treatment, p = 0.028, Mann Whitney test, but no significant change in GFAP and a trend of decrease in Fibronectin (unpaired Student’s t test). The astroglial scar size was also not significantly changed (unpaired Student’s t test). Scale bars: 100 µm (**b**), 50 µm (i and ii in **b**), and 500 µm (**c**).

At 1 dpi, SCI animals in both RGFP966- and vehicle-treated cohorts exhibited low BMS and TMS scores, confirming similar extent of injury (Fig. 4a). Subsequently, the RGFP966-treated animals exhibited improved hindlimb locomotion recovery compared to the vehicle cohort by both BMS and TMS scoring (p < 0.001, two-way ANOVA, Fig. 4a). Specifically, at 7 dpi, mice in the RGFP966-treated cohort began to show improved hindlimb function by BMS score. The improved functional recovery by RGFP966 treatment by 10 dpi was independently confirmed in three separate cohort studies (Supplementary Fig. 4). Effects of RGFP966 were sustained at 30 dpi, with the RGFP966 cohort reaching BMS scores between 3 and 4 (gravity bearing), while the BMS scores of the control cohort remained below 2 (Fig. 4a). No overt adverse effects of RGFP966 on animal health, including body weight, were observed (Supplementary Fig. 4). Of note, in the current study, we used a relative severe contusion SCI model, resulting in low BMS scores, precluding us from testing grid walk or foot print for functional recovery. Future studies using a milder contusion model would help to further assess the efficacy of RGFP966.

To further examine the basis of the beneficial effects of RGFP966 on SCI recovery, we assessed potential neuroprotective phenotypes of HDAC3 inhibition by comparing the number of axon fibers in the lesion core after contusion injury. RGFP966-treated animals displayed a significantly higher density of NF-H^+^ axon fibers in the lesion center than control mice at 10 dpi (p < 0.01, Fig. 4b). Given the relatively early stage of examination after SCI, the finding likely reflects axonal preservation rather than de novo regrowth. Microglia/macrophages can also influence functional states of fibroblasts and glia, e.g. regulating chondroitin sulfate proteoglycan (CSPG) in scar composition via expression of matrix degradation enzyme MMP-13^28^. We thus compared the size and constituency of the fibroglial scar at the injury site. We found that expression of CSPG was diminished by 56% in the RGFP966-treated group (p = 0.028, Mann Whitney test), while changes in fibronectin levels appeared more modest, and GFAP levels remained largely unaltered (Fig. 4c,d). The relative size of the astroglial scar was also comparable between the two cohorts (Fig. 4e). Of note, contusion injury resulted in a large variability in the fibroglial scar by these measures, thus affecting statistical values.

To further understand the basis of the broad repression of cytokine profiles by RGFP966, we investigated whether HDAC3 inhibition would affect transcription of inflammatory genes. qRT-PCR analyses on whole spinal cord tissue lysates at different time points after SCI did not reveal consistent changes between the two cohorts, which may reflect cell type diversity at the injury site. To specifically probe the microglia/macrophage population in the injured spinal cord, we turned to the Cx3cr1-CreER; INTACT mice to tag nuclei of macrophages and microglia. Of note, microglia are a self-renewing population with slow turnover^29^, whereas monocytes and macrophages have a faster turnover and are constantly replenished by bone marrow precursors that do not express Cx3cr1^30^. For our purposes, we administered tamoxifen during the peri-injury period to label both microglia and infiltrating macrophages (Supplementary Fig. 5). qRT-PCR of nuclear RNA extracted from the affinity-purified GFP^+^ nuclei confirmed an enrichment for microglia/macrophages (Supplementary Fig. 5). By transcriptional profiling, we revealed that HDAC3 inhibition resulted in broad suppression of immunity related genes (p < 0.01, two-way ANOVA, Supplementary Fig. 5). In accordance with the cytokine/chemokine antibody array data, expression of genes associated with both M1 state (e.g. *iNOS*, *Ciita* (MHCII)) and M2 state (*Cd163*, *Arg1*, *Vim*) appeared suppressed, further supporting a broad inflammatory suppression. Expression of cell surface markers was overall consistent with the IHC results: e.g. *Itgam* (CD11b) transcript levels were unchanged with RGFP966, in agreement with equal abundance of CD11b^+^ immune cells at the injury site between the two cohorts (see Supplementary Fig. 3). In addition, consistent with the IHC findings, transcript level of *Fcgr2b* (CD32) was decreased, whereas *Mrc1* (CD206) mRNA level was not markedly altered with RGFP966 treatment (Supplementary Fig. 5).

### HDAC3 activity does not directly affect axon growth potential of DRG neurons

**Figure 5:**
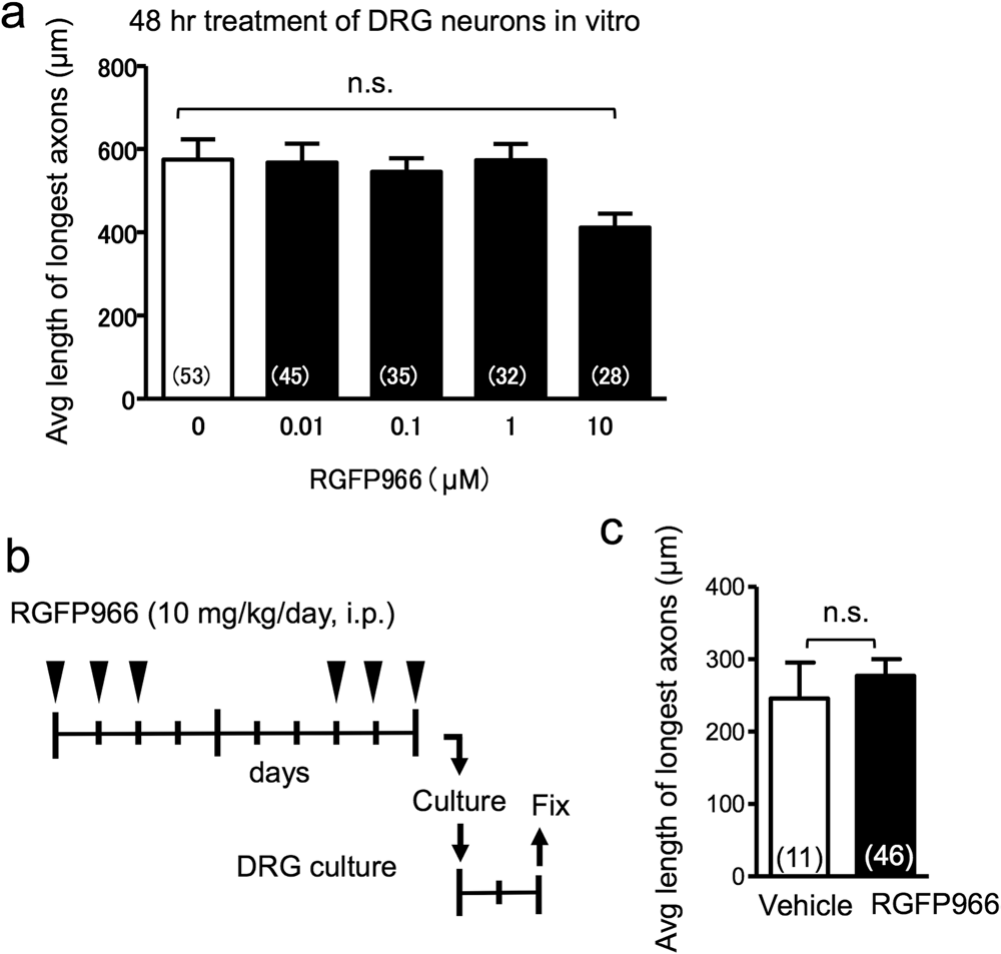
HDAC3 inhibition does not affect axon growth potential. (**a**) Dose-response neurite outgrowth assays show that RGFP966 treatment of cultured DRG neurons for 48 hr does not increase average length of longest axon. p = 0.073, Kruskal-Walis test. (**b**,**c**) Schematic diagram of *in vivo* RGFP966 treatment followed by DRG culture (**b**). No significant changes were observed for average axonal length between the two groups (**c**). unpaired Student’s t test. n.s. not statistically significant. The numbers of DRG neurons quantified are shown inside each bar graph.

The next question we wanted to address is whether the increased axon density at the injury site of the RGFP966-treated cohort may stem from a direct effect on axon growth of neurons. We first performed *in vitro* neurite outgrowth assays with semi-purified adult DRG neurons but found no evidence for increased axonal length by exposure to increasing doses of RGFP966 for 48 hr (Fig. 5a). In fact, at the highest dose of 10 µM, RGFP966 exposure resulted in a slight trend of decrease in neurite length (Fig. 5a). We also conducted further neurite outgrowth assays on adult DRG neurons isolated from animals that had received injection of RGFP966 or vehicle. Similarly, the axonal lengths were not significantly increased by *in vivo* RGFP966 treatment (Fig. 5b,c). Taken together, our results establish that the regenerative effects of HDAC3 inhibition after SCI are unlikely from a direct action on neuronal axon growth potential.

### HDAC3 activity regulates histone deacetylation in primary microglia upon inflammatory stimulation

**Figure 6:**
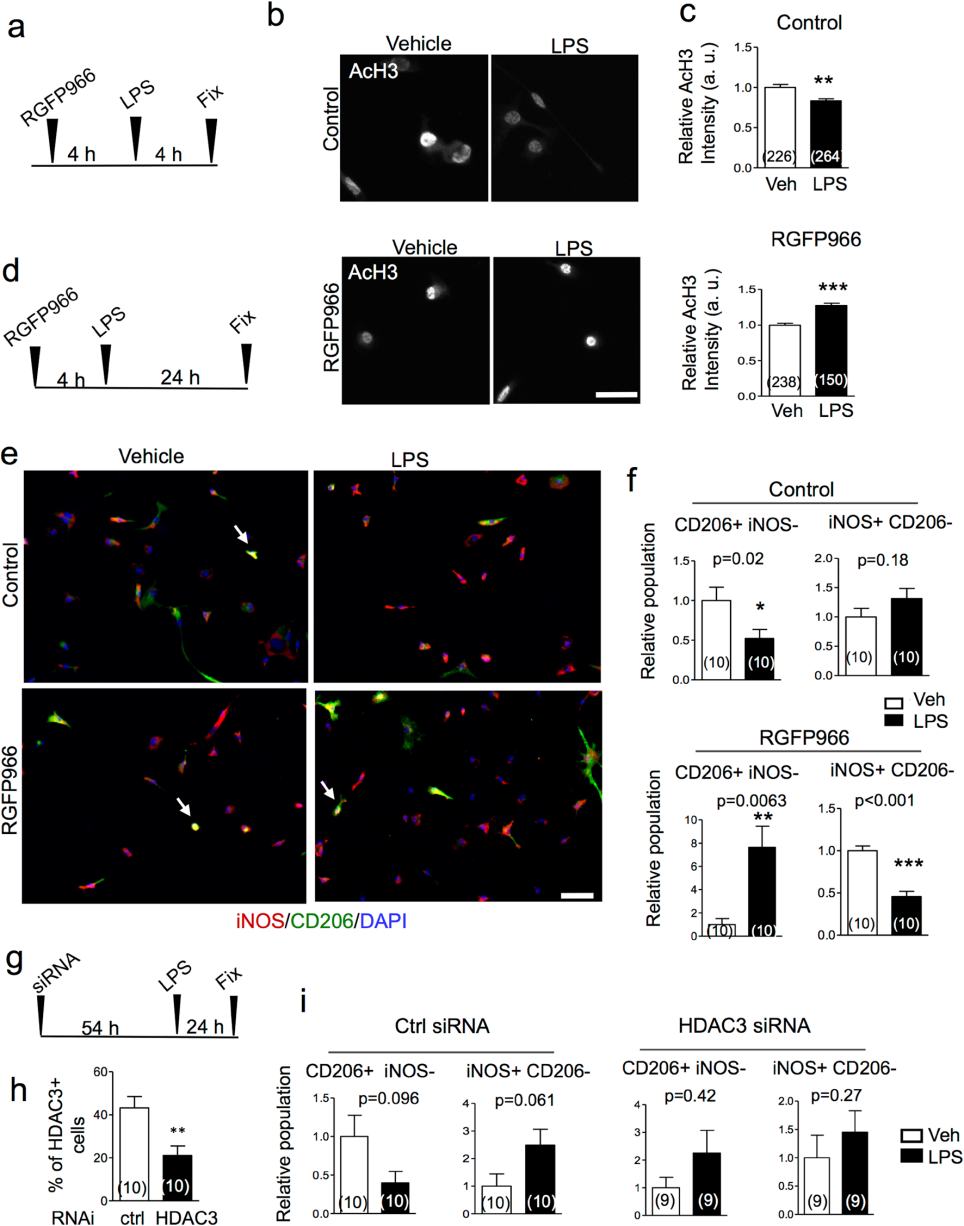
HDAC3 activity contributes to inflammatory activation of microglia. (**a**–**c**) Experimental scheme, representative images of immunocytochemistry, and quantification show that LPS stimulation results in reduced AcH3 levels in primary microglia by 4 hr. Pre-exposure to RGFP966 reverses LPS-triggered AcH3 reduction. The numbers inside the bar graph indicate the numbers of cells quantified from nine photographs for each experimental condition. Mann Whitney test. **p = 0.0079, ***p < 0.001. (**d**–**f**) Experimental scheme, representative images of immunocytochemistry and quantification show that LPS stimulation decreases CD206+ population (green) and a trend of increase in iNOS+ population (red). RGFP966 pre-treatment reversed the LPS-induced bias, leading to increased CD206+ population and deceased iNOS+ population. DAPI for nuclear counterstaining. Arrow: examples of double positive (iNOS+ CD206+) cells. The numbers inside the bar graph indicate the numbers of images quantified. Mann Whitey test and unpaired Student’s t test, respectively. (**g**–**i**) Experimental scheme and quantification show HDAC3 knockdown by siRNA in primary microglia (unpaired Student’s t test). HDAC3 knockdown abolishes the polarized responses of microglia to LPS stimulation. The numbers inside the bar graph indicate the numbers of images quantified by Mann Whitney tests. Scale bar: 25 µm (**a**) and 50 µm (**e**).

We next probed the impact of HDAC3 inhibition on microglial responses to classic inflammatory stimuli. Primary microglia were cultured to relative purity and then subjected to LPS or IL-4 stimulation to induce distinct phenotypes. First, we found that LPS stimulation resulted in reduced global levels of AcH3, evident at 4 hr after exposure to LPS (Fig. 6a–c), indicating rapid histone deacetylation in response to LPS. Pre-exposure to RGFP966, however, reversed the changes (Fig. 6a–c). Interestingly, the average AcH3 immunointensity was slightly increased by LPS with pre-exposure to RGFP966; it is conceivable that LPS stimulation may have increased both HDAC3 and HAT activities, the latter of which is unmasked with HDAC3 inhibition, leading to elevated AcH3 levels.

Next, we found that RGFP966 indeed altered inflammatory responses of microglia to LPS stimulation. Specifically, in vehicle controls, LPS stimulation resulted in a decrease in CD206^+^ iNOS^−^ cells (signifying an anti-inflammatory response) (p = 0.02), and a trend of increase in iNOS^+^ CD206^−^ population (signifying an inflammatory phenotype) (p = 0.18, Fig. 6d–f). RGFP966 pre-exposure reversed these changes upon LPS stimulation, resulting in an increase in CD206^+^ iNOS^−^ population (p < 0.01) and a decrease in iNOS^+^ CD206^−^ population (p < 0.001, Fig. 6d–f). Similar to the findings of AcH3, the reversal of the LPS-induced changes with RGFP966 might implicate both induced HDAC3 and HAT activities by LPS, the latter of which is unmasked with HDAC3 inhibition. It is also noteworthy that up to 20% of microglia co-expressed iNOS and CD206 (Fig. 6e), indicating that these canonical polarization markers are not exclusive for a particular activation state. Together, our data provide direct support for a central role of HDAC3 for regulating histone deacetylation, which enables LPS-induced inflammatory activation in microglia. RGFP966 did not further enhance IL-4-induced CD206 expression (data not shown), likely due to the already suppressed HDAC3 activity with IL-4 stimulation. To further establish HDAC3 as the main HDAC isoform in mediating inflammatory microglia activation, we performed HDAC3 knockdown by siRNA in primary microglia, which resulted in more than 50% cells displaying HDAC3 knockdown (Fig. 6g–h). Relative cell surface marker expression showed that the LPS-induced inflammatory activation of primary microglia was inhibited by HDAC3 siRNA (Fig. 6i).

## Discussion

Regenerative failure after SCI has been attributed to both a diminished intrinsic axon growth potential of mature CNS neurons and a hostile environment enriched in myelin-associated inhibitory molecules that hampers reparative efforts. Tackling either intrinsic or extrinsic barriers has yielded limited functional recovery, thus novel strategies based on different mechanisms are needed. Here, we reveal for the first time an HDAC3-mediated epigenetic regulation of the inflammatory gene network in microglia/macrophages after SCI. We demonstrate that inhibition of HDAC3 activity by a selective inhibitor, RGFP966, enables inflammatory suppression at the injury milieu, leading to neuronal protection/axonal preservation and improved functional recovery after SCI.

Contextual factors are the driving force for the diverse reactive states of microglia/macrophages after CNS injury. With a dominance of classic inflammatory cytokine IFNγ over Th2 cytokines IL-4 and IL-13 after SCI^14^, microglia and macrophages display a lingering inflammatory response, resulting in secondary injury, neurotoxicity, and impaired axonal regeneration^31^. In contrast, HDAC3 inhibition resulted in a decrease in IFNγ and TNFα, as well as a broad suppression of other cytokines/chemokines/acute phase proteins, leading to altered functionality of the innate immunity in favor of tissue repair after SCI.

The exact mechanism by which HDAC3 inhibition results in a broad suppression of inflammatory and acute phase responses in the setting of SCI remains to be determined. In injury models outside the CNS, HDAC3 has been shown to function as an epigenetic regulator of an inflammation-associated gene program^20^. Macrophages lacking HDAC3 are hypersensitive to IL-4 but less responsive to IFNγ stimulation^32^. This is consistent with our findings that cultured primary microglia no longer respond to LPS-stimulated inflammatory activation with HDAC3 inhibition. Future studies would be needed to identify specific gene loci that are under the direct regulation of HDAC3 in microglia/macrophages, in particular, residue-specific histone acetylation at inflammatory genes, such as H3K9ac (marking promoters of actively transcribed genes) and H3K27ac (marking enhancers that are functionally engaged in gene transcription). RGFP966 might also affect functions of other neural cell types. In addition, even though earlier pharmacokinetic studies have demonstrated adequate BBB penetration of RGFP966^21,22^, the *in vivo* efficacy and specificity of RGFP966 against HDAC3 in the CNS needs further validation. Using an immunoprecipitation-based fluorometric HDAC3 activity assay, we detected up to 50% inhibition of HDAC3 activity in brain tissues one hour after a single RGFP966 injection (10 mg/kg, i.p.); however, HDAC3 exists in large protein complexes, including other HDACs, and moreover, RGFP966 is a competitive, reversible inhibitor of HDAC3, which may limit the detection sensitivity of the IP-based assay. Future studies using conditional ablation of HDAC3 in microglia and macrophages will provide genetic evidence and delineate cell-type-specific function of HDAC3 in the context of SCI. Furthermore, microglia and peripherally derived macrophages may exert slightly different functions. Recent studies demonstrated divergent spatial distributions, with microglia surrounding injury center and macrophages concentrating at lesion core after SCI^33,34^. Whether HDAC3 plays distinct roles in these two populations awaits future studies.

It has been postulated that a major deleterious aspect of the innate immune response in post-SCI setting is a lingering inflammatory phenotype that does not resolve towards a pro-repair state, unlike in non-CNS injuries. In our current study, RGFP966 was administered during the acute phase after SCI; as such, both microglia and macrophages were exposed to RGFP966 from an early time point on, thereby affecting the injury milieu from the very beginning after injury. The early changes in injury milieu may influence functional states of the immune cells at later stages even though RGFP966 concentrations may have already waned. It would be of great interest to determine in future studies whether HDAC3 inhibition at later phases would result in similar changes of local cytokine profile at the already established injury sites. It is also noteworthy that SCI results in a breach of blood brain barrier (BBB), which may facilitate higher RGFP966 concentration at the injury site as compared to uninjured CNS tissue. Likewise, it is noteworthy of the highly dynamic nature of the injury milieu with changing immune cell populations and shifting cytokine profiles during the multiphasic immune response. For instance, earlier study showed that the number of CD16/32^+^ macrophages increases between 1 and 14 dpi after SCI, reaching ~60% of macrophages, while the fraction of CD206^+^ cells peaks at ~8% of macrophages and declines to 3% by 14 dpi^19^. Thus, infiltrating macrophages are in various reactive states contingent upon their arrival time and settlement in specific niches in the injury microenvironment.

The full implication of a global suppression of inflammatory and acute phase responses in influencing cellular and behavioral outcomes needs further clarification. The pro-repair functions of microglia/macrophages are likely multifactorial, including scar reduction by secretion of MMP13^28^, increased remyelination via promotion of oligodendrocyte differentiation^35^, or neurotrophic effects^36^. In addition, macrophages may also promote axonal regeneration/sprouting by secreting growth-enhancing factors or reducing inhibitory molecules such as CSPG. It is worth discussing that in the current study, the increased axonal density in the lesion center was observed at 10 dpi, a relatively early time point after SCI, thus it likely reflects neuroprotective effects of HDAC3 inhibition, and not de novo axonal regeneration per se. This is further supported by the fact that improved BMS scores were already evident at 7 dpi, when it is unlikely that newly regenerated fibers have reached targets and are integrated into neural circuits. In fact, neuronal protection and axonal preservation/sprouting may be mainly affecting local interneurons, which can induce microcircuit reconstruction and improve functional recovery^37^. Additionally, identifying key neuromodulatory factors secreted by microglia/macrophages in the context of HDAC3 inhibition will further shed light in this matter. Our results extend the repertoire of critical functions of HDAC3 in mediating tissue homeostasis and disease progression^38^. The current study focused on the histone deacetylase function of HDAC3. HDACs can also deacetylate non-histone targets and exert enzyme-independent effects^39^, it is thus conceivable that some of the observed effects of HDAC3 inhibition may be independent of histone deacetylation.

Our data also highlights the emerging disconnect of the M1/M2 polarization model with the *in vivo* gene signatures and functional states of the innate immune cells after CNS injury. In injured spinal cord, we found that simultaneous expression of CD16/32 and CD206 in individual cells was frequently detected. Likewise, cultured microglia contained up to 20% of iNOS^+^ CD206^+^ double-positive cells. Hence, these canonical polarization markers are not exclusive for a particular activation state, thus conceptualization of the diverse functional states of the innate immune cells requires a complete update.

Immunomodulation can be combined with strategies that boost intrinsic axon growth potential to achieve optimal functional recovery in SCI. In this context, it is worth mentioning that our previous studies revealed a regeneration promoting effect of MS275, an inhibitor of HDAC1, on enhancing the intrinsic regeneration capacity of DRG neurons through global elevation of AcH4 and induction of multiple regeneration-associated genes (RAGs)^40^. Intriguingly, different HDACs likely exert distinct functions in different cell types in SCI. Combined manipulation of specific HDAC isoforms may thus lead to favorable chromatin landscapes in a cell-type specific manner to achieve maximal functional recovery after SCI.

## Materials and Methods

### Spinal cord injury

All animal procedures were approved and performed in accordance with the guidelines and protocols approved by the Institutional Animal Care and Use Committee at the Icahn School of Medicine at Mount Sinai. Five- to 7-week-old C57BL/6J female mice (Jackson Laboratory) were used for spinal cord injury models. For dorsal column transection model, after T8 laminectomy, the dorsal column was bilaterally transected using iris microscissors (Fine Science Tools) as described previously^41^. For contusion injury, after T8 laminectomy, an infinite horizon impactor (IH-0400, Precision Systems and Instrumentation) was used to deliver a force of 70 kDyn. RGFP966 (10 mg/kg, Selleckchem) or vehicle was prepared as described^21^ and administered by intraperitoneal injection at 2, 24, and 48 hr after spinal cord injury. Fluorometric HDAC3 activity assay was performed using kits from Sigma (catalog #EP1013) and BPS Bioscience (catalog #50073). Following the contusion injury, motor function of each hindlimb was measured by Basso mouse scale score (BMS) (scale of 0–8 points)^26^ and Toyama mouse score (TMS) (scale of 0–30 points)^42^ every day in a blinded fashion. One mouse showed a BMS score of 3 at 1 day after the injury, and was thus excluded; while the rest of the mice all scored between 0–2 by BMS after the contusion injury. All animals received subcutaneous injection of 1 ml of saline, 10 mg/kg of Baytril and 0.05 mg/kg of buprenorphine daily for the first week following surgery.

### Immunohistochemistry

Spinal cords were fixed with 4% paraformaldehyde (PFA), cryoprotected in 30% sucrose, embedded in OCT, and sagittally sectioned (10 μm thickness). The sections were blocked with 5% normal donkey serum or 5% normal goat serum in 0.3% Triton-X-PBS and immunostained for CD11b (1:300, MBS420973, MyBioSource, or 1:500, 14–0112, Affymetrix), Iba1(1:150, NB100–1028, Novus), histone deacetylase 1 (HDAC1, 1:500, ab19845, Abcam), HDAC2 (1:500, ab16032, Abcam), HDAC3 (1:100, sc-11417, Santa Cruz), HDAC8 (1:50, sc-11405, Santa Cruz), CD16/32 (1:50, 553142, BD Pharmingen), CD206 (1:500, AF2535, R&D Systems), acetylated histone 3 (AcH3, 1:500, 06–599, Millipore), acetylated histone 4 (AcH4, 1:1000, 06–866, Millipore), neurofilament-H (NF-H, 1:1000, AB5539, Millipore), chondroitin sulfate proteoglycan (CSPG, 1:100, clone CS56, C8035, Sigma), glial fibrillary acidic protein (GFAP, 1:1000, clone GA5, MAB360, Millipore), and fibronectin (1:80, ab2033, Millipore). Subsequently, AlexaFluor 488-, AlexaFluor 594-, and/or DyLight 405-conjugated secondary antibodies (1:300, Jackson ImmunoResearch or Invitrogen) were used. DAPI was used for counterstaining. Images were captured with Zeiss microscopes (AxioCamMRc or Axiocam 503 mono). Several images around injury center were captured in each section, and automatically combined with Photoshop (Adobe) or Zen (Zeiss) software. One to 4 sections close to midline were captured for each spinal cord. The images were analyzed with MetaMorph (Molecular Devices) or ImageJ (National Institutes of Health) and averaged per mouse.

### Microglia culture

Microglial cells were cultured as described^43^ with modifications. Cerebral cortices of postnatal day 2 (P2) C57BL/6 mice were dissected and treated with 0.25% trypsin (Invitrogen) for 30 min at 37 °C. After washing with 10% fetal bovine serum (FBS)-DMEM/F-12 (Invitrogen), the cells were treated with 600 U/ml DNase and 0.3 mg/ml trypsin inhibitor (Invitrogen) for 15 min at 37 °C. A 15% BSA cushion was used to remove debris by centrifugation. The cells were cultured for ~2 weeks on a 6-cm dish coated with 5 μg/ml poly-D-lysine (PDL). Four days after reaching confluence, the cells were treated with 0.25% trypsin diluted 1:3 in DMEM/F-12 for 1 hr at 37 °C. After washing the dish, only microglia remained attached on the dish. The cells were detached with 0.25% trypsin and replated on 8 well slides (Falcon) coated with 5 μg/ml PDL. Cells were treated with 10 μM RGFP966 for 4 hr and subsequently 0.1 μg/ml lipopolysaccharide (LPS, Sigma) for 4 or 24 hr. For knockdown, 100 nM HDAC3 siRNA (ON-TARGETplus SMARTpool siRNA, J-043553–05, J-043553-06, J-043553-08, J-043553-17, Dharmacon) or control siRNA was transfected into the cells with DharmaFECT 4 (Dharmacon). Fifty-four hr after transfection, the cells were treated with 0.1 μg/ml LPS and examined 24 hr later.

After the LPS treatment, the cells were fixed with 4% PFA supplemented with 4% sucrose, blocked with 3% bovine serum albumin and 1% normal donkey serum in 0.3% Triton-X-PBS, and immunostained for acetylated H3 (1:500), iNOS (1:100, 610328, BD Biosciences), CD206 and HDAC3. Subsequently, AlexaFluor 488- and AlexaFluor 594-conjugated secondary antibodies were used. DAPI was used for counterstaining. Ten images were captured for each treatment, and analyzed with MetaMorph.

### Dorsal root ganglion neuron culture

Dorsal root ganglion (DRG) neurons were cultured as described previously^40^. The DRG neurons were treated with increasing doses of RGFP966 for 48 hr, and fixed and immunostained for β-tubulin III (1:500, clone TUJ1, MAB1195, R&D systems), and counterstained with DAPI. Lengths of the longest axon per neuron were measured with ImageJ and averaged. DRG neurons were also isolated from adult mice treated by intraperitoneal injection of RGFP966 (10 mg/kg) or vehicle at the indicated days before the cell culture and fixed after 48 hr of culture.

### Cytokine Array

Ten days after contusion injury, injured regions were collected and protein was extracted with Illustra triplePrep Kit (GE healthcare). 100 µg of protein lysate were used for the cytokine array (Proteome Profiler antibody assays-Mouse Cytokine Array Panel A, R&D Systems). Images were captured with a Gel Doc XR system (Bio-Rad), and analyzed with ImageJ.

### Statistical analysis

For each experiment, the numbers of mice used in each cohort, and the number of images analyzed from each animal are listed in figure legends and Supplementary Table 1. For each data set, Shapiro-Wilk test (IBM SPSS statistics software, version 23) was performed to determine parametric (p> 0.05) vs. non-parametric samples (p< 0.05 in the Shapiro-Wilk test). For parametric data, F-test was conducted to compare variances (Prism 5, GraphPad Software, La Jolla, CA). For samples with similar variance (p > 0.05 in the F-test), unpaired t-test (Prism 5) was used. For samples with significantly different variances (p < 0.05 in the F-test), unpaired t-test with Welch’s correction (Prism 5) was performed. For statistical comparison between non-parametric vs. parametric data sets, or between 2 non-parametric data sets, Mann-Whitney test was performed, which does not require the assumption of normal distributions (Prism 5). For statistical comparison among multiple non-parametric data sets, Kruskal-Walis test was performed. For studies with repeated measures, two-way ANOVA repeated measures (RM) followed by post hoc Bonferroni test were performed. Cluster-based summary statistics using within-subject averaging were performed whenever possible, and Split-plot ANOVA was performed for multilevel analysis of nested data with small sample size. Outliers were excluded based on Grubbs’ outlier test (Graphpad, p < 0.05). The mean values are presented along with standard error of the mean (SEM) as error bars.

## Electronic supplementary material

Supplementary data
