# Author Correction: HDAC3 inhibition ameliorates spinal cord injury by immunomodulation

**DOI:** 10.1038/s41598-018-25249-3

**Published:** 2018-04-27

**Authors:** Tomoharu Kuboyama, Shalaka Wahane, Yong Huang, Xiang Zhou, Jamie K. Wong, Andrew Koemeter-Cox, Michael Martini, Roland H. Friedel, Hongyan Zou

**Affiliations:** 1 Fishberg Department of Neuroscience, Friedman Brain Institute, Icahn School of Medicine at Mount Sinai, New York, New York, 10029 USA; 2 Department of Neurosurgery, Friedman Brain Institute, Icahn School of Medicine at Mount Sinai, New York, New York, 10029 USA; 3 Division of Neuromedical Science, Institute of Natural Medicine, University of Toyama, Toyama, 930-0194 Japan; 4 Present Address: Tisch MS Research Center of New York, New York, New York, 10019 USA; 5 Present Address: Alzheimer’s Drug Discovery Foundation New York, New York, 10019 USA

Correction to: *Scientific Reports* 10.1038/s41598-017-08535-4, published online 17 August 2017

In the Supplementary Information file originally published with this Article, an additional dataset containing a list of sample sizes, distributions, variances and statistical methods and Supplementary Experimental Procedures was omitted. These errors have been corrected in the Supplementary Information that now accompanies the Article.

