# Supplementary material for: HDAC3 inhibition ameliorates spinal cord injury by immunomodulation
Source: Sci Rep. 2017 Aug 17;7:8641. doi: 10.1038/s41598-017-08535-4 (PMC5561061; doi:10.1038/s41598-017-08535-4)
Supplement: Supplementary file 1 — Supplementary data [file 41598_2017_8535_MOESM1_ESM.pdf]

HDAC3 inhibition ameliorates spinal cord injury by immunomodulation

Tomoharu Kuboyama [1,3], Shalaka Wahane [1], Yong Huang [1], Xiang Zhou[1], Jamie K. Wong [1, 4], Andrew Koemeter-Cox [1], Michael Martini [1], Roland H. Friedel [1,2], and Hongyan Zou [1, 2, #]

[1] Fishberg Department of Neuroscience and [2] Department of Neurosurgery, Friedman Brain Institute, Icahn School of Medicine at Mount Sinai, New York, New York 10029, USA
[3] Division of Neuromedical Science, Institute of Natural Medicine, University of Toyama, Toyama 930-0194, Japan
[4] Current address: Tisch MS Research Center of New York, New York, New York 10019, USA

[#]Correspondence should be addressed to:
Email: hongyan.zou@mssm.edu

Contents:

Supplementary Figures 1-5
Supplementary Table 1
Supplementary Experimental Procedures

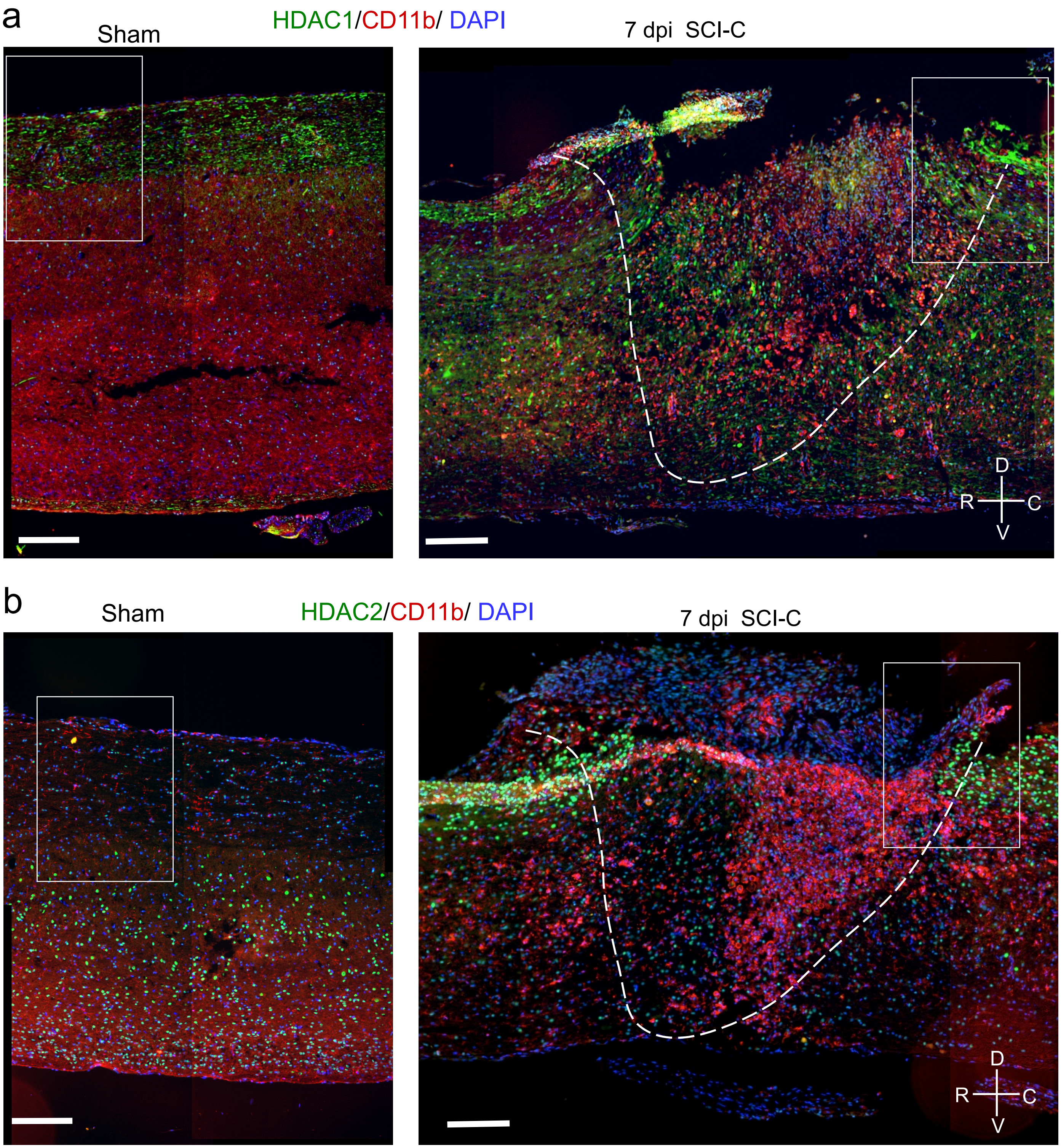

Figure S1: Distinct expression patterns of class I HDACs after SCI. (**a-b**) Representative immunohistochemistry images with co-immunolabeling of the indicated HDACs (green), CD11b (red), and nuclear counterstaining by DAPI (blue) show distinct expression dynamics of class I HDACs at the injury site 7 days after contusion (7 dpi SCI-C). Orientation: R: Rostral, C: Caudal, D: Dorsal, V: Ventral. Scale bar: 200 μm (a-d). Enlarged images in boxed area are shown in (**e**).

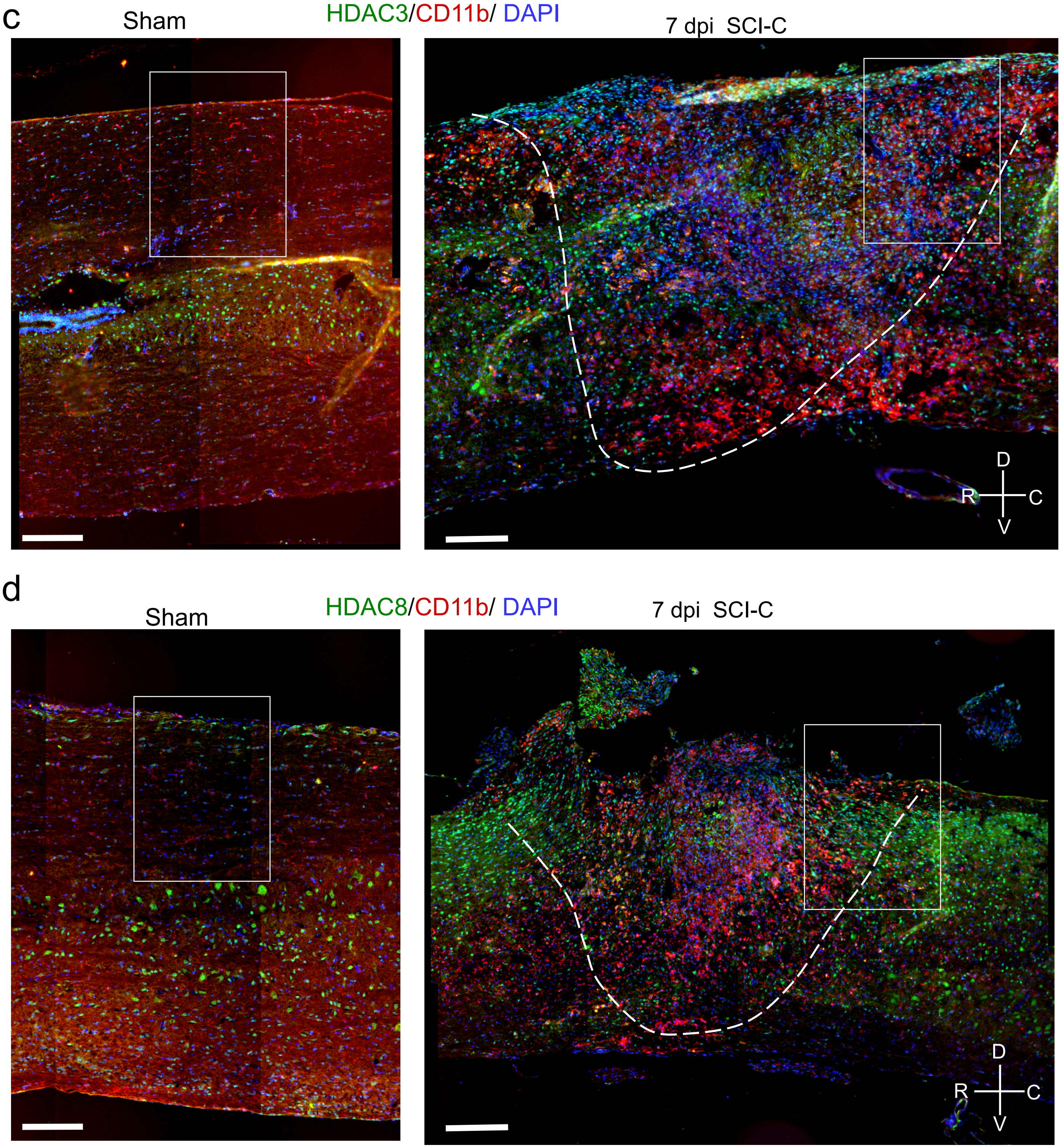

Figure S1: Distinct expression patterns of class I HDACs after SCI. (**c-d**) Representative immunohistochemistry images with co-immunolabeling of the indicated HDACs (green), CD11b (red), and nuclear counterstaining by DAPI (blue) show distinct expression dynamics of class I HDACs at the injury site 7 days after contusion (7 dpi SCI-C). Orientation: R: Rostral, C: Caudal, D: Dorsal, V: Ventral. Scale bar: 200 μm (a-d). Enlarged images in boxed area are shown in (**e**).

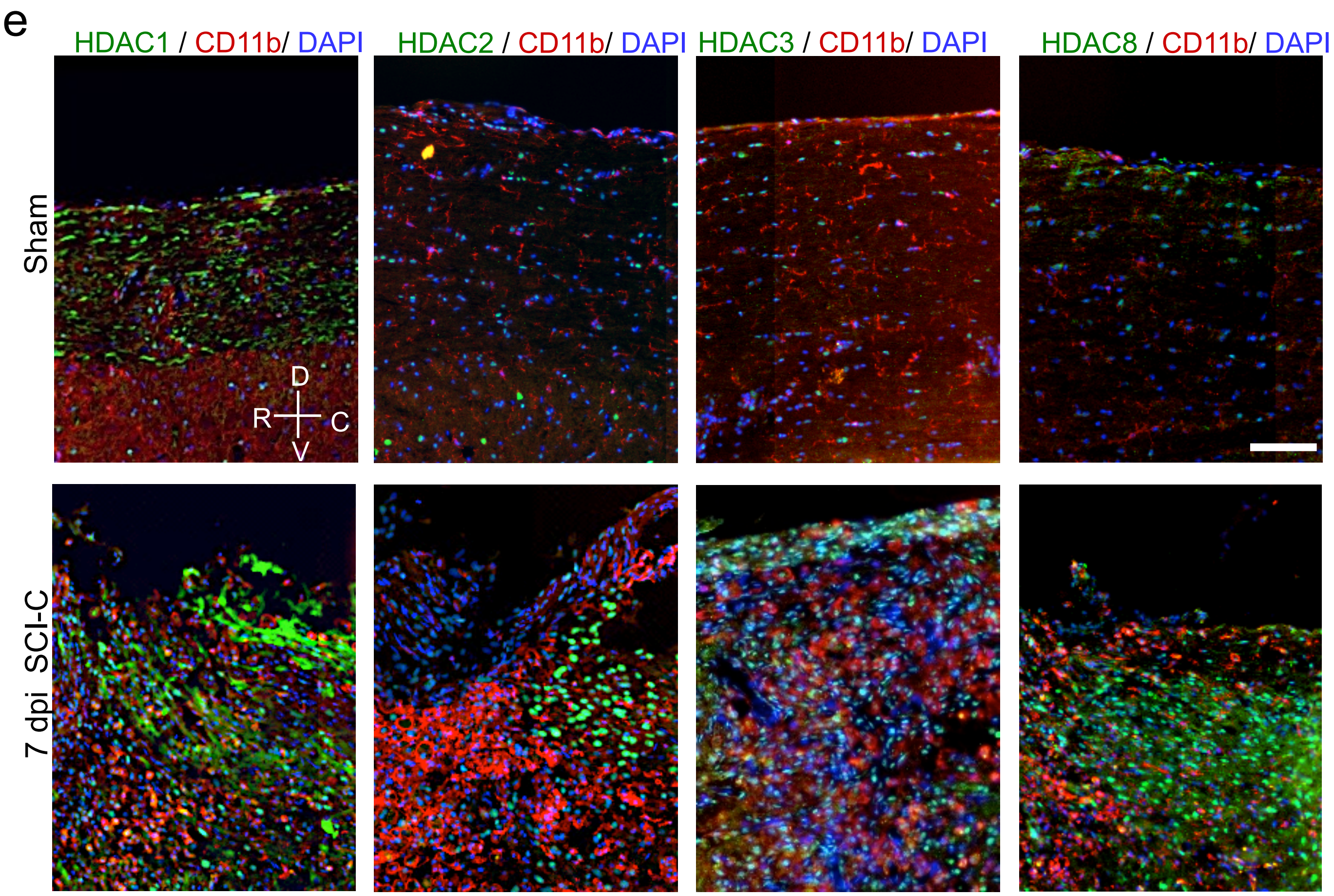

Figure S1: Distinct expression patterns of class I HDACs after SCI. (e) Enlarged images of the boxed area in (a-d) of IHC co-immunolabeling showing that only HDAC3 largely overlaps with CD11b after SCI. Orientation: R: Rostral, C: Caudal, D: Dorsal, V: Ventral. Scale bar: 100 μm (e). For higher magnification images, please refer to Fig. 1c-d.

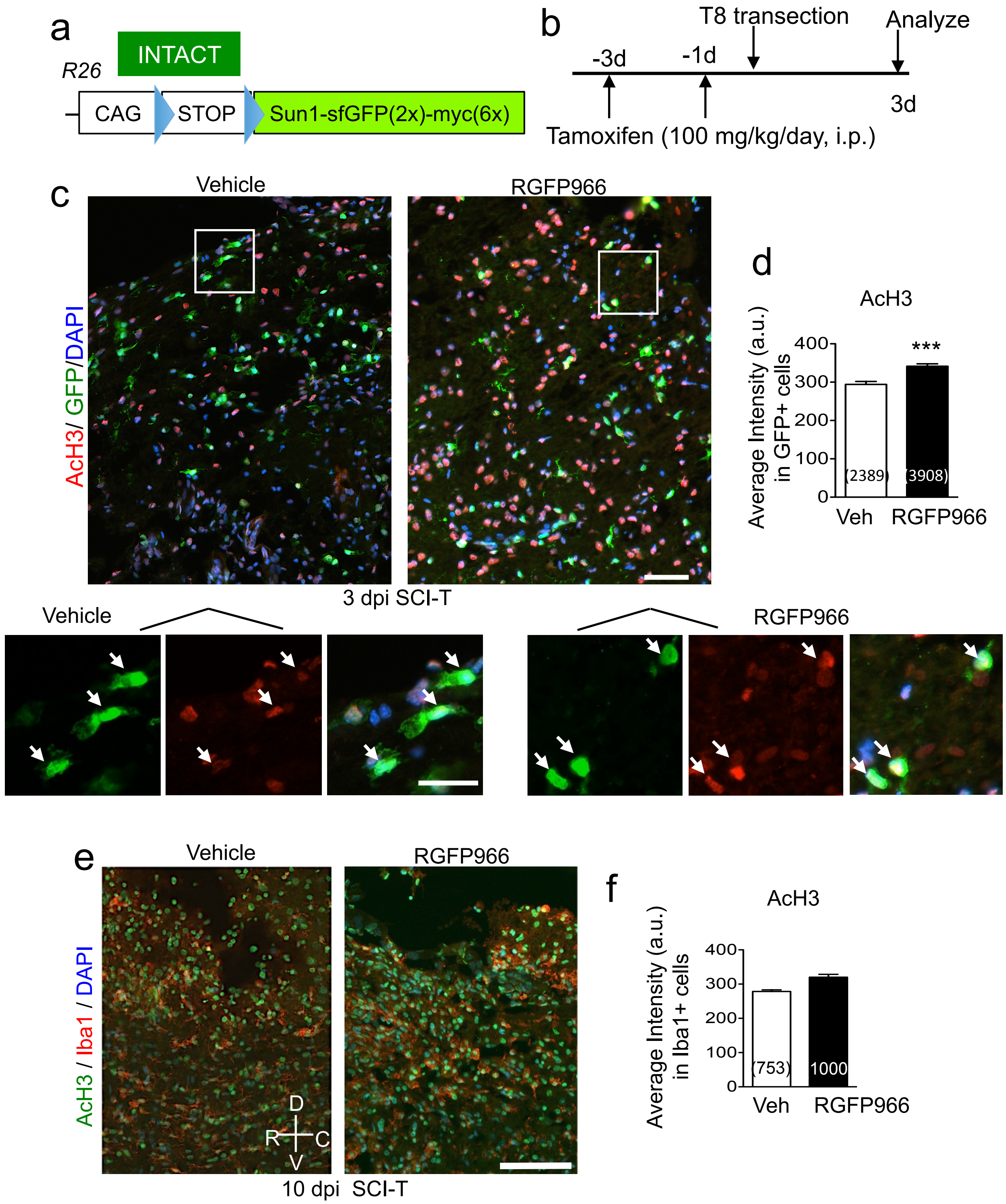

Figure S2: RGFP966 increases global histone H3 acetylation levels in spinal cord at 3 dpi. (a) Diagram of INTACT allele for conditional expression of GFP-tagged Sun1 from the Rosa26 locus. **(b)** Tamoxifen regimen to label microglia and macrophages after SCI. **(c-d)** Representative IHC images and quantification showing increased average levels of AcH3 in GFP+ immune cells at the injury site at 3 dpi in INTACT; Cx3Ccr1^{CreER} mice by RPGF966. ***, $p < 0.001$, Mann Whitney test. **(e-f)** Representative IHC images show levels of AcH3 (green) in Iba1+ (red) immune cells at the injury site at 10 dpi after T8 dorsal column transection (SCI-T). The number of Iba1+ cells quantified from 4 or 5 animals in each cohort and 3 images for each animal are shown inside the bar graph, Mann Whitney test, $p > 0.05$. Scale bar: 50 μm (c), 100 μm (e) and 25 μm (enlarged images in c).

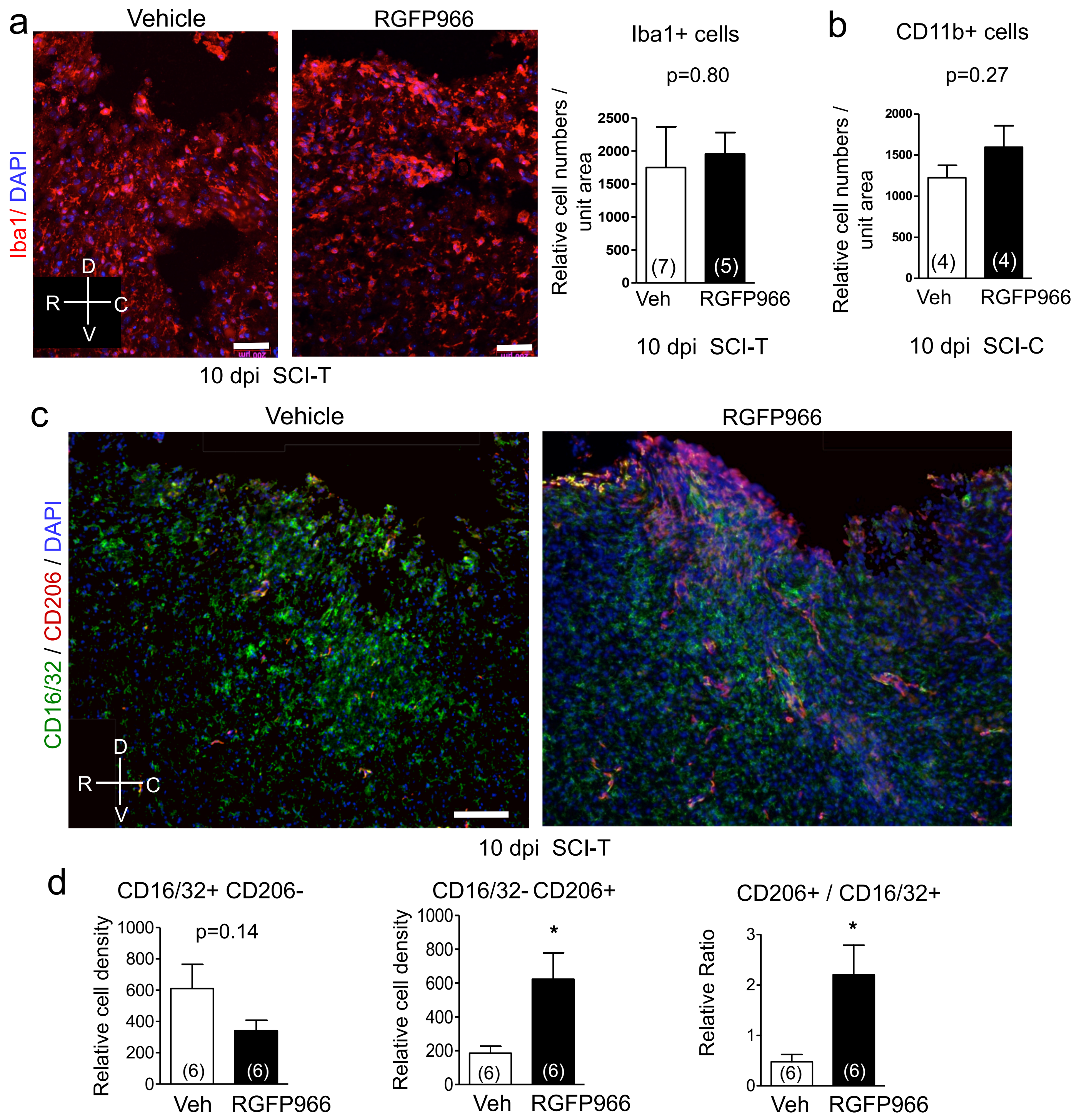

Figure S3: HDAC3 inhibition alters functional state of the innate immune cells after SCI. (a) Representative images and quantification show comparable number of Iba1+ cells at the injury site at 10 dpi after T8 dorsal column transection (SCI-T) in vehicle and RGFP966 cohorts. unpaired Student's *t*-test, p=0.80. (**b**) Quantification shows no significant changes in the number of CD11b+ cells at the injury site at 10 dpi after contusion (SCI-C). unpaired Student's *t*-test, p=0.27. **(c-d)** Representative images and quantification show the numbers of CD16/32+ macrophages (green), CD206 macrophages (red), and the relative ratio at the injury site at 10 days after T8 dorsal column transection (SCI-T). DAPI for nuclear counterstaining. unpaired Student's *t*-test, with Welch correction for the latter two comparisons. *, p<0.05. Orientation of the spinal cord, R: Rostral, C: Caudal, D: Dorsal, V: Ventral. The number of animals tested are indicated inside the bar graph, with 2-3 representative image averaged for each animal. Scale bar: 50μm (**a** and **c**).

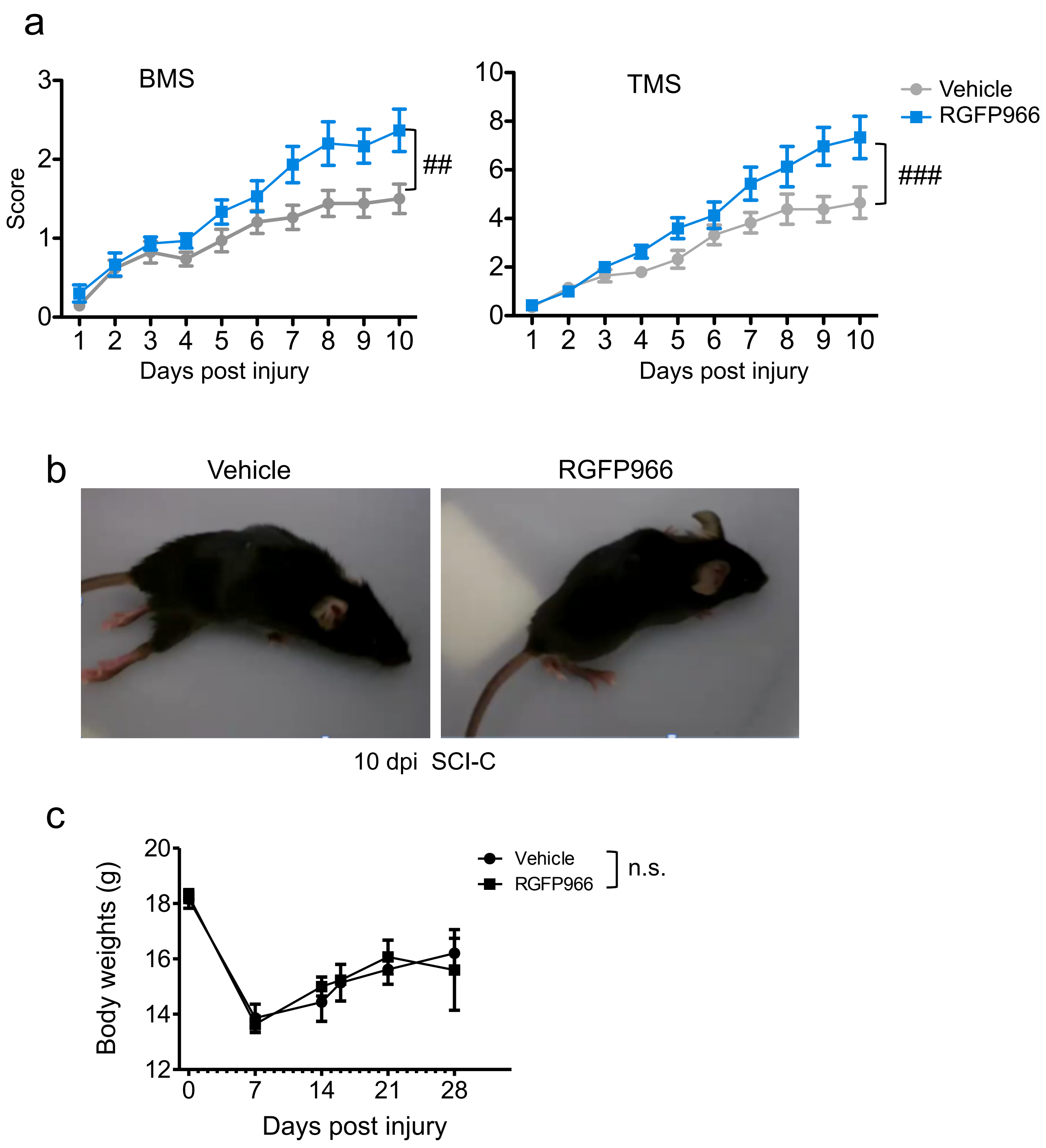

Figure S4: HDAC3 inhibition improves functional recovery after SCI. **(a)** Behavioral assays from three independent experiments demonstrate improved functional recovery with RGFP966 by both BMS and TMS after contusion injury. ##, $p<0.01$; ###, $p<0.001$, repeated measures, two-way ANOVA with Bonferroni post hoc correction. *, $p<0.05$; **, $p<0.01$. Data were compiled from three independent experiments, with a total of 34 mice in vehicle cohort and 30 mice in RGFP966 cohort. **(b)** Representative images of mice at 10 days post-contusion demonstrate that vehicle-treated animal often display hindlimb paralysis. In contrast, RGFP966-treated animals frequently display weight-bearing function of hindlimbs. **(c)** Body weights were comparable between vehicle and inhibitor treated cohorts. $p>0.05$, repeated measures two-way ANOVA. n.s., not statistically significant difference.

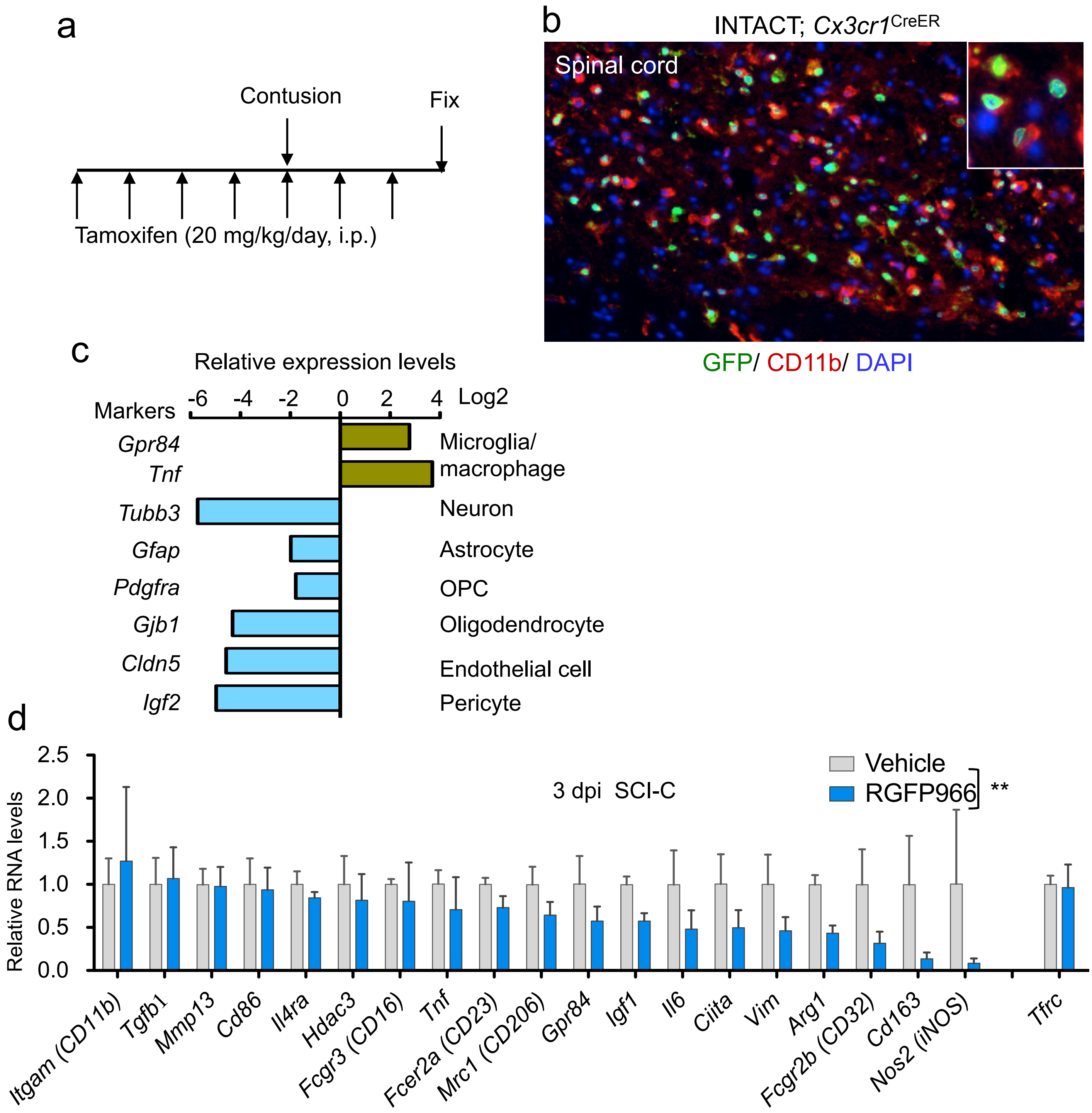

Figure S5: HDAC3 inhibition results in a broad suppression of inflammatory genes in microglia and macrophages after SCI. **(a)** Schematic diagram of tamoxifen pulsing to label microglia and infiltrating macrophages after contusion SCI. **(b)** Immunohistochemistry image of injured spinal cord of INTACT; *Cx3cr1*^{CreER} mice at 3 dpi after contusion. GFP tagged nuclei (green) overlap with CD11b^{+} immune cells (red). DAPI for nuclear counterstaining. **(c)** qRT-PCR of nuclear RNA from affinity purified nuclei with anti-GFP antibodies show enrichment of microglia/macrophages by relative expression levels of cell type-specific markers as normalized to housekeeping gene *Tfrc*. **(d)** Relative expression levels of the indicated genes by qRT-PCR of INTACT-purified nuclear RNA of microglia/macrophages from injured spinal cord at 3 dpi after contusion. Results were normalized to housekeeping gene *Gapdh* and averaged (n=3 pairs of mice). **, $p<0.01$, two-way ANOVA with Bonferroni post hoc correction. Housekeeping gene *Tfrc* showed no change.

Supplementary Table 1. List of sample size, distribution, variences and statisticical methods.

Data sets for two group comparison — Statistical method used for each data set

Figure	n (number of animals used in each cohort)	Shapiro-Wilk test to determine parametric / non-parametric data	Sample distribution	F-test to compare variances	Sample variances	unpaired t-test	Unpaired t-test with Welch's correction	Mann Whitney test	Multilevel Analysis Nested data
Fig. 1a HDAC3+ cells	3,3 mice, 3 images from each mouse, total 9 images per each condition	P = 0.250 (sham), 0.522 (7 dpi SCI-T)	parametric	P < 0.0001	unequal variances		Unpaired t-test with Welch's correction, P < 0.0001		parametric split-plot ANOVA, p = 0.0150, F(1, 4) = 16.7196
Fig. 2b % of HDAC3	3,3 mice, 3 images from each mouse, total 9 images per each marker	P = 0.514 (CD16/32), 0.363 (CD206)	parametric	P = 0.0012	unequal variances		Unpaired t-test with Welch's correction, P = 0.0001		parametric split-plot ANOVA, p = 0.0240, F(1, 4) = 12.5216
Fig. 2d AcH3	3,3 mice, 3 Images per animal, 3029 CD16/32+ cells, 6245 CD206+ cells	P = 0.00 (CD16/32), 0.00 (CD206)	non-parametric					Mann Whitney p < 0.0001	
Fig. 2d AcH4	3,3 mice, 3 Images per animal, 1687 CD16/32+ cells, 2305 CD206+ cells	P = 0.00 (CD16/32), 0.00 (CD206)	non-parametric					Mann Whitney p < 0.0001	
Fig. 3f CD16/32+CD206- cells	8,8 mice, 2-4 images from each mouse	P = 0.594 (vehicle), 0.315 (RGFP966)	parametric	P = 0.8081	equal variances	Unpaired t-test p=0.025			
Fig. 3f CD206+ CD16/32- cells	8,8 mice, 2-4 images from each mouse	P = 0.490 (vehicle), 0.327 (RGFP966)	parametric	P = 0.0126	unequal variances		Unpaired t-test with Welch's correction, P = 0.16		
Fig. 3f ratio of CD206+/CD16/32+	8,8 mice, 2-4 images from each mouse	P = 0.287 (vehicle), 0.044 (RGFP966)	non-parametric	P < 0.0001	unequal variances		Unpaired t-test with Welch's correction, p = 0.073		
Fig. 4b NF-H	3,4 mice, 1-4 image(s) from each mouse	P = 0.422 (vehicle), 0.222 (RGFP966)	parametric	P = 0.3495	equal variances	unpaired t-test, p<0.01			
Fig. 4d CSPG	8,8 mice, 2-4 images from each mouse	P = 0.026 (vehicle), 0.409 (RGFP966)	non-parametric					Mann Whitney p = 0.028	
Fig. 4d GFAP intensity	4,5 mice, 3-5 images from each mouse	P = 0.792 (vehicle), 0.057 (RGFP966)	parametric	P = 0.1113	equal variances	unpaired t-test, p=0.60			
Fig. 4d fibronectin	8,8 mice, 2-4 images from each mouse	P = 0.096 (vehicle), 0393 (RGFP966)	parametric	P = 0.1533	equal variances	unpaired t-test, p=0.092			
Fig. 4e GFAP area	8,8 mice, 2-4 images from each mouse	P = 0.607 (vehicle), 0.704 (RGFP966)	parametric	P = 0.9181	equal variances	Unpaired t-test, p=0.086			
Fig. 5c neurite length in vivo	11,46 neurons, 10,26 photos	P = 0.088 (vehicle), 0.540 (RGFP966)	parametric	P = 0.7379	equal variances	unpaired t-test, p>0.05			
Fig. 6c AcH3 Control	226, 264 cells, 9,9 photos	P = 0.00 (vehicle), 0.00 (RGFP966)	non-parametric					Mann Whitney p = 0.0079	
Fig. 6c AcH3 RGFP	238, 150 cells, 9,9 photos	P = 0.00 (vehicle), 0.009 (RGFP966)	non-parametric					Mann Whitney p < 0.0001	
Fig. 6f Cont CD206+	10,10 photos	P = 0.082 (vehicle), 0.777 (RGFP966)	parametric	P = 0.2324	equal variances	unpaired t-test, p=0.02			
Fig. 6f Cont iNOS+	10,10 photos	P = 0.370 (vehicle), 0.316 (RGFP966)	parametric	P = 0.6640	equal variances	unpaired t-test, p=0.18			
Fig. 6f RGFP CD206+	10,10 photos	P = 0.000 (vehicle), 0.198 (RGFP966)	mix of parametirc and non-parametric					Mann Whitney p = 0.0063	
Fig. 6f RGFP iNOS+	10,10 photos	P = 0.116 (vehicle), 0.221 (RGFP966)	parametric	P = 0.7633	equal variances	unpaired t-test, p<0.001			
Fig. 6h % HDAC3	10,10 photos	P = 0.568 (control siRNA), 0.786 (HDAC3 siRNA)	parametric	P = 0.5765	equal variances	unpaired t-test, p<0.01			
Fig. 6i siCont CD206+	10,10 photos	P = 0.345 (vehicle), 0.022 (LPS)	non-parametric					Mann Whitney p =0.096	
Fig. 6i siCont iNOS+	10,10 photos	P = 0.04 (vehicle), 0.395 (LPS)	non-parametric					Mann Whitney p = 0.061	
Fig. 6i siHDAC3 CD206+	9,9 photos	P = 0.016 (vehicle), 0.024 (LPS)	non-parametric					Mann Whitney p = 0.42	
Fig. 6i siHDAC3 iNOS+	9,9 photos	P = 0.001 (vehicle), 0.265 (LPS)	non-parametric					Mann Whitney p = 0.27	
Sup Fig. 2d AcH3 RGFP at 3 dpi	4,4 mice, 3-7 images per animal, outlier excluded by Grubbs outlier test, 2389,3908 GFP+ cells	P = 0.00 (vehicle), 0.00 (RGFP966)	non-parametric					Mann Whitney p<0.0001	
Sup Fig. 2f AcH3 levels at 10 dpi	4,5 mice, 1 Image per animal, 753,1000 Iba1+ cells,	P = 0.00 (vehicle), 0.00 (RGFP966)	non-parametric					Mann Whitney, p >0.05	
Sup Fig. 3a Iba1+	7,5 mice, one represenative photo from each mouse	P = 0.257 (vehicle), 0.431 (RGFP966)	parametric	P = 0.1384	equal variance	unpaired t-test, p=0.80			
Sup Fig. 3b CD11b+	4,4 mice, 2-3 images from each mouse	P = 0.606 (vehicle), 0.579 (RGFP966)	parametric	P = 0.3857	equal variance	unpaired t-test, P = 0.27			
Sup Fig. 3d CD16/32+	6,6 mice, 2-3 images from each mouse	P = 0.15 (vehicle), 0.293 (RGFP966)	parametric	P = 0.0900	equal variance	unpaired t-test, P = 0.14			
Sup Fig. 3d CD206+	6,6 mice, 2-3 images from each mouse	P = 0.122 (vehicle), 0.571 (RGFP966)	parametric	P = 0.0106	unequal variance		Unpaired t-test with Welch's correction, p = 0.042		
Sup Fig. 3d CD206/CD16/32	6,6 mice, 2-3 images from each mouse	P = 0.052 (vehicle), 0.664 (RGFP966)	parametric	P = 0.0078	unequal variance		Unpaired t-test with Welch's correction, p = 0.036		

Data set with more than 2 groups

Fig. 3g, cytokine array	4,4 mice			2 way RM ANOVA, with Bonferroni post hoc correction			
Fig. 4a and Sup Fig. 4a, BMS, TMS score	10, 6 mice, and 34, 30 mice			2 way RM ANOVA, with Bonferroni post hoc correction			
Fig. 5a, in vitro neurite length		P = 0.00, 0.046, 0.241, 0.006, 0.394	mixed	p = 0.073 (Kruskal-Walis test)			
Sup Fig. 4c body wt	10, 6 mice			2 way RM ANOVA, with with Bonferroni post hoc correction			
Sup Fig. 5d RT-PCR	3, 3 mice			2 way ANOVA with Bonferroni post hoc correction.			

Supplementary Experimental Procedures

Mouse mutants

$R26^{INTACT}$ mice [B6;129-Gt(ROSA)26Sor$^{tm5(CAG\text{-}SUN1/sfGFP)Nat}$/J] [1] were crossed with $Cx3cr1^{CreER}$ mice [B6.129P2(Cg)-Cx3cr1$^{tm2.1(Cre/ERT)Litt}$/WganJ] [2] to generate $R26^{INTACT/+}$; $Cx3cr1^{CreER/+}$ mice (both lines from the Jackson Laboratory).

Quantitative RT-PCR of INTACT-RNA

$R26^{INTACT/+}$; $Cx3cr1^{CreER/+}$ mice of mixed gender received daily intraperitoneal tamoxifen injections (20 mg/kg/day) for 7 days. On day 5, the mice were subjected to contusion spinal cord injury. RGFP966 (10 mg/kg) or vehicle was intraperitoneally administered at 2, 24, and 48 hr after injury. Three days after injury, spinal cords around injured region were collected and frozen in liquid nitrogen. Immunopurification of microglia/macrophage-specific nuclei was performed and nuclear RNA was isolated as described [1]. The nuclear RNA from 2 or 3 spinal cords was pooled, converted to cDNA and amplified with Ovation RNA-Seq System V2 (NuGEN) per manufacturer's protocol. Three pooled samples for each experimental condition were compared. Quantitative PCR was performed using 5 ng of the amplified cDNA for each reaction with PerfeCTa SYBR Green FastMix Rox (QuantaBiosciences) in an ABI 7900HT qPCR system (Applied Biosystems). Housekeeping genes *Tfrc* or *Gapdh* were used for normalization. Sequences of primers are listed below: *Gpr84* forward: CTCCTGCTACCATGAGTCTGT; *Gpr84* reverse: GTGCAGTAGAGTAGATCAGCCA, *Tnf* forward: CCCTCACACTCAGATCATCTTCT; *Tnf* reverse: GCTACGACGTGGGCTACAG, *Tubb3* forward: GTCTCTAGCCGCGTGAAGTC; *Tubb3* reverse: GCAGGTCTGAGTCCCCTACA, *Gfap* forward: CACGAACGAGTCCCTAGAGC; *Gfap* reverse: GTAGGTGGCGATCTCGATGT, *Pdgfra* forward: TGGCATGATGGTCGATTCTA; *Pdgfra* reverse: CGCTGAGGTGGTAGAAGGAG, *Gjb1* forward: ACAGCCATTGGCCGAGTATG; *Gjb1* reverse: TGTTGGTGAGCTACGTGCATT, *Cldn5* forward: GCAAGGTGTATGAATCTGTGCT; *Cldn5* reverse: GTCAAGGTAACAAAGAGTGCCA, *Igf2* forward: GTGCTGCATCGCTGCTTAC; *Igf2* reverse: ACGTCCCTCTCGGACTTGG, *Itgam* forward: ATGGACGCTGATGGCAATACC; *Itgam* reverse: TCCCCATTCACGTCTCCCA, *Tgfbi* forward: CAGCACGGCCCCAATGTAT; *Tgfbi* reverse: GGGACCTTTTCATATCCAGGACA, *Mmp13* forward: GGAAGACCTTGTGTTTGCAGAGC; *Mmp13* reverse:

CACTGTAGACTTCTTCAGGATTCCCG, *Cd86* forward: TTGTGTGTGTTCTGGAAACGGAG; *Cd86* reverse: AACTTAGAGGCTGTGTTGCTGGG, *Il4ra* forward: GGATAAGCAGACCCGAAGC; *Il4ra* reverse: ACTCTGGAGAGACTTGGTTGG, *Hdac3* forward: TTGGTATCCTGGAGCTGCTT; *Hdac3* reverse: GACCCGGTCAGTGAGGTAGA, *Fcgr3* forward: TTGCTTTTGCAGACAGGCAGA; *Fcgr3* reverse: TTCGCACATCAGTGTCACCAT, *Fcer1g* forward: CAGCTATAGCCAGCCGTGAG; *Fcer1g* reverse: ACTGGGGTGGTTTCTCATGC, *Mrc1* forward: GTGGTCCTCCTGATTGTGATAG; *Mrc1* reverse: CACTTGTTCCTGGACTCAGATTA, *Igf1* forward: TGGATGCTCTTCAGTTCGTG; *Igf1* reverse: GCAACACTCATCCACAATGC, *Il6* forward: ACTTCACAAGTCGGAGGCTT; *Il6* reverse: TGCAAGTGCATCATCGTTGT, *Ciita* forward: GACGCTCAACTTGTCCCAAAAC; *Ciita* reverse: GCAGCCGTGAACTTGTTGAAC, *Vim* forward: AGCTAACCAACGACAAAGCC; *Vim* reverse: TCCACTTTGCGTTCAAGGTC, Arg1 forward: CATTTGGGTGGATGCTCACA; *Arg1* reverse: TGGTACATCTGGGAACTTTCCTTT, *Fcgr2* forward: AATCCTGCCGTTCCTACTGATC; *Fcgr2* reverse: GTGTCACCGTGTCTTCCTTGAG, *Cd163* forward: TGCTGTCACTAACGCTCCTG; *Cd163* reverse: TCATTCATGCTCCAGCCGTT, Nos2 forward: ACCAAGCTGAACTTGAGCGA; Nos2 reverse: GCCCCATAGGAAAAGACTGC, *Trfc* forward: TGGGAACAGGTCTTCTGTTG; *Trfc* reverse: TGCAGTCCAGCTGGCAAAGA, *Gapdh* forward: AGGTCGGTGTGAACGGATTTG; *Gapdh* reverse: TGTAGACCATGTAGTTGAGGTCA.

References

1. Mo, A., *et al.* Epigenomic Signatures of Neuronal Diversity in the Mammalian Brain. *Neuron* **86**, 1369-1384 (2015).
2. Parkhurst, C.N., *et al.* Microglia promote learning-dependent synapse formation through brain-derived neurotrophic factor. *Cell* **155**, 1596-1609 (2013).
